# A temperature-tuned electrochemiluminescence layer for reversibly imaging cell topography†

**DOI:** 10.1039/d2sc04944a

**Published:** 2022-11-10

**Authors:** Cheng Ma, Zejing Xing, Xiaodan Gou, Li-Ping Jiang, Jun-Jie Zhu

**Affiliations:** School of Chemistry and Chemical Engineering, Yangzhou University Yangzhou 225002 P. R. China; State Key Laboratory of Analytical Chemistry for Life Science, School of Chemistry and Chemical Engineering, Nanjing University Nanjing 210023 P. R. China jianglp@nju.edu.cn jjzhu@nju.edu.cn

## Abstract

Investigating electrochemiluminescence (ECL) scenarios under different temperatures is important to expand its imaging scope near an electrode surface. Here, we develop a temperature-tuned ECL layer by recording the evolution of shadow regions of adherent cells. Finite element simulation and experimental results demonstrate that the thickness of the ECL layer (TEL) is reversibly regulated by electrode temperature (*T*_e_), so that single cell topography at different heights is imaged. The TEL in two ECL routes shows different regulation ranges with elevated *T*_e_, thus providing a flexible approach to adjust the imaging scope within specific heights. In addition, a heated electrode significantly improves the image quality of cell adhesion in heterogeneous electrochemical rate-determined situations. Thus, the contrast in cell regions shows a reversible response to *T*_e_. This work provides a new approach to regulate the TEL and is promising for monitoring transient heat generation from biological entities.

## Abstract 2

A temperature-tuned electrochemiluminescence layer is designed for reversibly imaging single cell topography. The regulated thickness range of the ECL layer depends on the adopted ECL routes, namely the catalytic route and oxidative reduction route.

## Graphical abstract

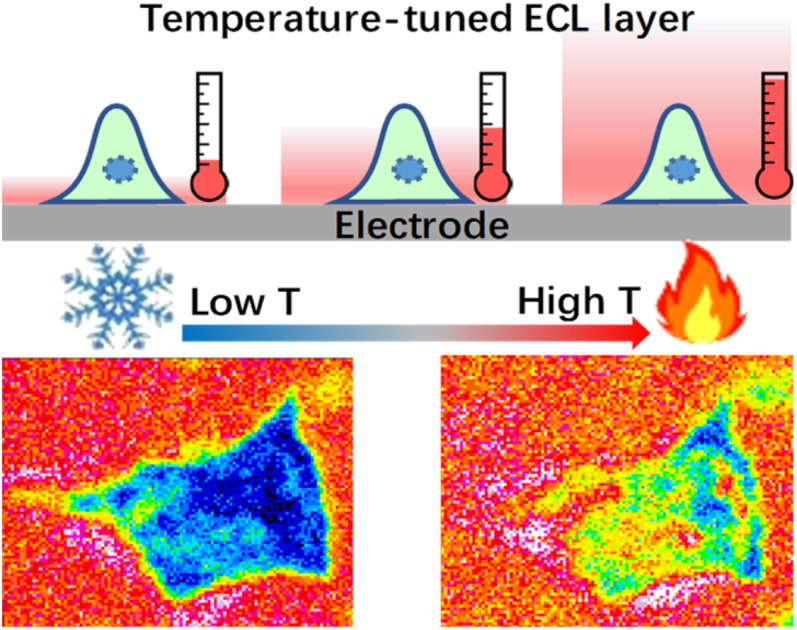

## Introduction

Thermoelectrochemical studies have attracted much attention because of their value in obtaining accurate thermodynamic data and promoting practical performance in sensing, electrocatalysis and batteries.^1–3^ Because temperature is an important factor for tuning electrochemical processes, elevating electrode temperature (*T*_e_) generally lowers overvoltage, accelerates the reaction rate, and promotes mass transport.^4–6^ These effects especially benefit electroanalytical systems involving heterogeneous electrode reactions with coupled homogeneous chemical reactions in the vicinity of the electrode (*e.g.*, proton-coupled electron transfer (PCET) and electrochemiluminescence (ECL)).^7–10^ Although many reports focused on the design of high-temperature electrochemical setups for better sensitivity and reproducibility, it remains challenging to *in situ* visualize the electron-transfer rates and electrogenerated species transport as a function of *T*_e_.

ECL is an electrochemically triggered light generated by electron transfer reactions between electrogenerated radical intermediates.^11–16^ Therefore, the luminescence feature in ECL processes can directly reflect the electrochemical scenario during elevating *T*_e_.^17^ Although high temperature reduced ECL quantum efficiency in the ion annihilation pathway,^18^ most coreactant-based ECL systems showed noticeably enhanced luminescence under heated electrodes that accelerated electrochemical rates and species transport *via* diffusion and convection.^7,19,20^ As a result, various analytical indicators such as sensitivity, stability and reproducibility were significantly improved with elevated *T*_e_. Nevertheless, quantitatively measuring the relationship between temperature and the ECL emission layer is still challenging and remains to be explored.

In past few decades, people have made much effort to develop excellent methods of optical section, including confocal laser scanning microscopy (CLSM),^21–23^ multiphoton microscopy,^24–26^ total internal reflection fluorescence (TIRF),^27,28^ light-sheet microscopy^29–31^ and optical sectioning structured illumination microscopy (OS-SIM).^32–34^ Among them, CLSM and multiphoton microscopy have been viewed as the most useful techniques to get 3D tomography images from single cells to thick tissue samples. However, they bear the disadvantages of photobleaching and phototoxic damage.^35,36^ In addition, the depth of the illuminated layer in TIRF is too shallow (<200 nm)^37^ and a sophisticated setup is acquired for light-sheet microscopy and OS-SIM. Therefore, an integration of label-free, thickness tunable and convenient installation is a desirable requirement for the optical section technique. ECL imaging is more efficient in minimizing the background signals for the sensitive measurement because ECL is a kind of chemiluminescence triggered by electrochemical reactions. Therefore, ECL imaging can avoid the photobleaching, photothermal effect, and light scattering effects. Recently, optical microscopy has provided a visualized tool to directly observe ECL phenomena including the electron transfer rate at the electrode–solution interface and species diffusion in the vicinity of the electrode.^38–42^ The versatile microscopy technique has been used ranging from ECL mechanism discussion to biological applications.^43–46^ For example, the thickness of the ECL layer (TEL) was depicted by ECL microscopy,^44^ which was further used for not only the selective imaging of cell–matrix and cell–cell junctions but also the investigation of the chemical lens effect.^46,47^ Thanks to the perfect match between the surface-confined ECL layer and vertical dimensions of single biological entities, ECL microscopy highlighted its advantages of imaging cell adhesion, mitochondria and basal membrane proteins with high vertical resolution.^40,48–52^ The imaging feature is similar to total internal refection fluorescence microscopy (TIRFM), while the TEL is primarily controlled by the lifetime of the oxidized luminophore and coreactant radicals. Because the vertical imaging scope of ECL microscopy is largely determined by its TEL, exploring new strategies to regulate the TEL is critical for expanding the application range of the ECL technique.

**Fig. 1:**
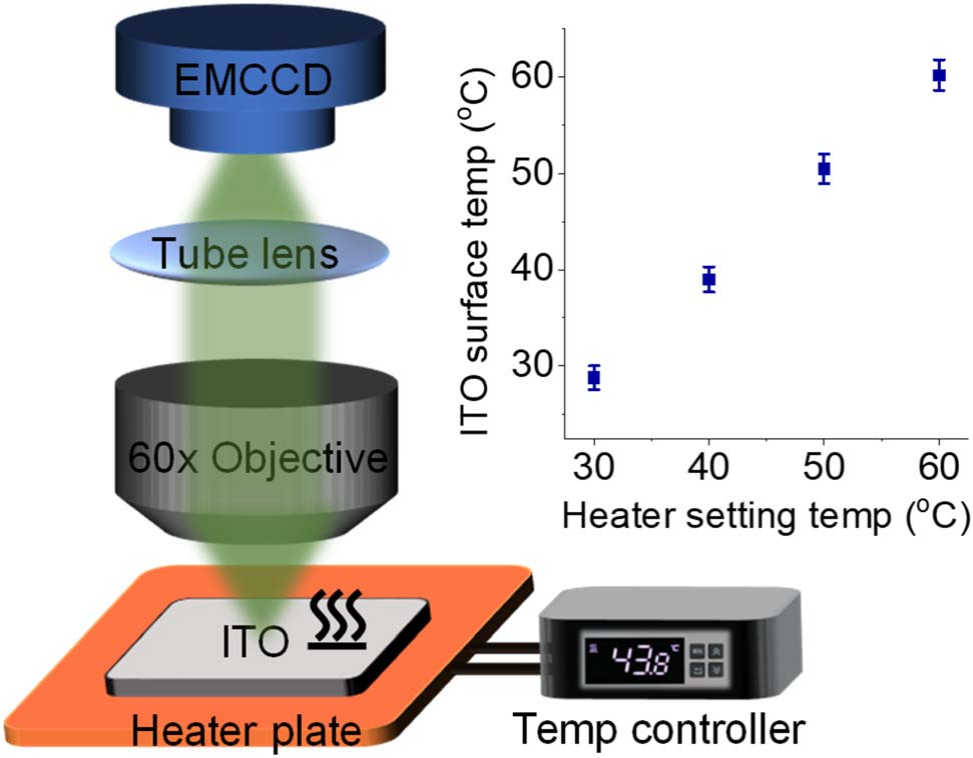
Schematic illustration of the ECL microscope coupled with a heated ITO electrode, heater plate, and digital temperature controller. Inset shows the consistency between heater setting temperature and ITO surface temperature measured by using an infrared thermometer.

**Fig. 2:**
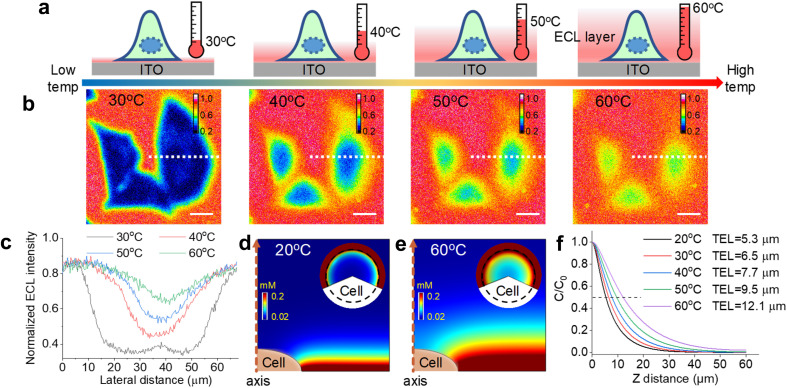
(a) Schematic illustration of ECL layer evolution with elevated *T*_e_ from 30 °C to 60 °C. The electrode surface is modified with an attached cell. The ECL layer gradually thickens with elevated *T*_e_. (b) Shadow ECL patterns of three cells at different *T*_e_ from 30 °C to 60 °C. The ECL intensity at each pixel was normalized. Scale bar (white) is 20 μm. (c) Normalized ECL intensity profiles across the cell (white dashed line in (b)) at different *T*_e_. (d and e) Simulated ECL layer, namely Ru(bpy)_3_^2+^* distribution, from the side view at 20 °C and 60 °C, respectively. Inset: circular panels show simulated cellular ECL patterns from the top view and the dashed circle denotes the cell region. (f) Normalized ECL intensity along the vertical direction from the electrode surface to solution with elevated *T*_e_. These profiles along the vertical direction and TEL at different *T*_e_ are simulated results from COMSOL. Electrolyte is 200 mM PBS (pH 7.0) containing 1 mM Ru(bpy)_3_^2+^ and 10 mM TPrA. The anodic voltage is 1.3 V *vs.* Ag/AgCl.

Herein, temperature-tuned ECL microscopy is developed for the generation of an adjustable ECL layer, which enables fine and reversible regulation of the TEL for imaging cell topography at different heights (Fig. 1 and 2). Under diffusion-controlled conditions, high *T*_e_ significantly increases the TEL due to the accelerated diffusion rate of electrogenerated products near the electrode surface. Simulated results demonstrate the possibility of optical sectioning by the difference between adjacent temperature-dependent TELs. In addition, we discover the different regulation ranges of the TEL under the oxidative reduction route and catalytic route. Therefore, elevating *T*_e_ shows different ECL imaging scenarios of adherent cells under the two ECL routes. In addition to the regulated TEL, the imaging sensitivity is significantly improved with a heated electrode due to the effect of temperature on electrode kinetics, leading to clearer cell contours at low anodic potential or low luminophore concentration. Because no systematic studies concerning temperature-tuned ECL layers have been explored, we anticipate that the combination between a heated electrode and ECL microscopy provides a new tool for observing the thermo-ECL phenomenon and performing more thermo-electrochemical applications.

## Results and discussion

To couple a heated electrode with an ECL microscope, we used a digital heater plane that was closely attached to the non-conductive side of the ITO electrode (Fig. 1), leaving the conductive side for ECL imaging. An infrared thermometer confirmed the good controllability of *T*_e_ by the heated device (Fig. S1†). Then the classic Ru(bpy)_3_^2+^/TPrA system was employed because its ECL scenario is related to electrochemical and photophysical processes including heterogeneous electron transfers, homogeneous reactions and ECL emission yield (Scheme S1†). Each of these steps can be regulated by the temperature of systems.^17,18^ While the ECL emission yield of Ru(bpy)_3_^2+^ decreased with raising temperature in ion annihilation reactions,^18^ the heated electrode accelerated the electrochemical oxidation rates and diffusion rates of Ru(bpy)_3_^2+^ and TPrA, eventually resulting in an enhanced ECL intensity in coreactant systems.^17^ It is possibly explained by the enhanced electrochemical rates overwhelming the decline in the quantum yield of excited Ru(bpy)_3_^2+^*. Because of the spatial and temporal resolution, ECL microscopy can provide access to direct visualization of the temperature-tuned ECL layer and relevant ECL evolution beyond room temperature.

For adherent cells, the different TELs offered spatially selective imaging of cells at different heights.^46^ Therefore, a homemade upright microscope was built to record ECL images of adherent cells on the conductive side of the ITO electrode (Fig. 1). Due to the volume exclusion effect of insulated cells, cellular regions showed shadow ECL contrast compared with blank regions (Fig. 2a and b).^49,53^ Because the shape of cellular shadow ECL was influenced by the TEL,^46^ we recorded the evolution of cellular shadow ECL with elevated *T*_e_. At 30 °C, the periphery of the dark ECL pattern was similar to the cellular outlines in the dark-field image (Fig. S2†), indicating a very thin ECL layer near the electrode surface. In this case, only the cellular bottom parts within the thin ECL layer were imaged. As *T*_e_ increased, however, cellular shadow patterns gradually shrunk from the adhesion periphery to the cellular center (Fig. 2b). Because the adherent cell has a roughly plano-convex shape, the disappeared shadow at the cell periphery suggests the remarkable extension of the ECL layer in the vertical direction.^46^ To exclude the influence from defocus and thermal drift caused by the thermal expansion of the ITO electrode, the cells were refocused after *T*_e_ was stabilized over five minutes. Also, the extended TEL was confirmed by the decreased contrast between cellular and blank regions where ECL contrast denoted the difference of normalized ECL intensity.^46^ The ECL contrast of the cellular center region was significantly weakened from 0.49 ± 0.02 to 0.20 ± 0.02 when the temperature raised to 60 °C (Fig. 2c and S3†). Here, ECL contrast is the difference value of normalized ECL intensity between cellular center regions and blank regions. To exclude the changes of the cell morphology and topology under different temperatures, we recorded the dark-field images of cells. Fig. S4† shows that the cell morphology and topology remained unchanged with elevating temperature from 30 °C to 60 °C.

To rationalize the fine regulation of the TEL by *T*_e_, finite element simulations were conducted. First, the diffusion coefficients of ECL reagents at different temperatures were obtained by using Randles–Sevcik and Stokes–Einstein equations (see COMSOL simulation in the ESI†). According to eqn (S7),† the TEL is determined from the concentration profiles of Ru(bpy)_3_^2+^*, which is the reaction product of the oxidant Ru(bpy)_3_^3+^ and reductant TPrA radical (TPrA˙) (eqn (S5)†). Because high temperature generally improves diffusion coefficients, the diffusion distance of Ru(bpy)_3_^3+^ is extended away from the electrode surface (Fig. S8†). Here the dominant ECL reactions follow the catalytic route due to the high concentration ratio of Ru(bpy)_3_^2+^ and TPrA. Accordingly, the concentration profile of Ru(bpy)_3_^3+^ is responsible for the homogeneous generation of TPrA˙ (eqn (S6) and (S3)†). Fig. S9† shows that TPrA˙ gradually extends into solution with elevated temperature. This TPrA˙ away from the electrode surface is primarily generated by homogeneous reactions rather than a heterogeneous electro-oxidation reaction due to its short life time. Although higher temperature also accelerates heterogeneous electrochemical reactions, simulated results show that the concentration profiles of Ru(bpy)_3_^2+^ are totally determined by the diffusion coefficient at mass-transfer controlled potential (1.3 V *vs.* Ag/AgCl) (Fig. S10†). In other words, the increased diffusion rates rather than heterogeneous electrode kinetics of Ru(bpy)_3_^2+^ should cause the thickened ECL layer at such a high anodic potential.

The side view of the ECL layer demonstrates the increased TEL with elevated temperature (Fig. S11†). At each *T*_e_, the concentration profile of Ru(bpy)_3_^2+^* always shows a gradually decreasing trajectory (Fig. 2f). However, elevating *T*_e_ makes the ECL intensity descend less steeply in the vertical direction. If the TEL is defined as the full width at half maximum of Ru(bpy)_3_^2+^* concentration, then the TEL is 5.3 μm, 6.5 μm, 7.7 μm, 9.5 μm, and 12.1 μm at 20 °C, 30 °C, 40 °C, 50 °C, and 60 °C, respectively. For example, at 20 °C the TEL is about the half height of the cell from the side view, which produces a clear cell contour from the top view (Fig. 2d). However, at 60 °C the ECL layer nearly overpasses the whole cell so that cellular edges disappear from the top view (Fig. 2e). In addition, the insets in Fig. 2d and e show that ECL contrast at the cell center is weakened from the top view with elevated *T*_e_. These simulated results at different *T*_e_ are consistent with the experimental images captured with the ECL microscope.

**Fig. 3:**
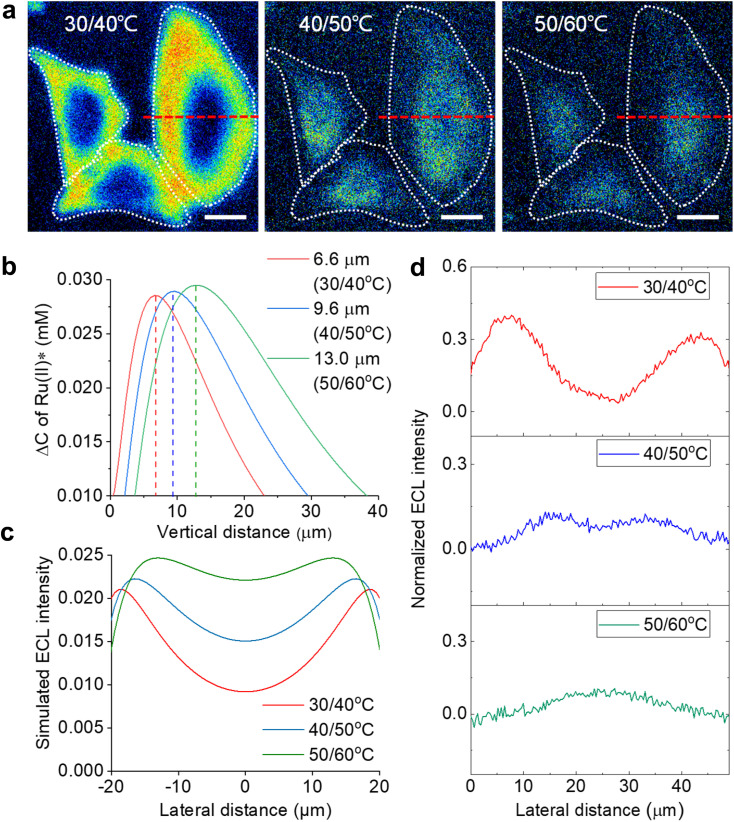
(a) ECL section of cells by image subtraction under two adjacent *T*_e_. The white dashed lines denote the cellular edges. Scale bar (white) is 20 μm. (b) The simulated vertical distribution of the Ru(bpy)_3_^2+^* difference between two adjacent electrode temperatures. (c) Simulated lateral ECL profiles over the cell from the top view in ECL section imaging. (d) Experimental ECL profiles along the red dashed lines in (a). Electrolyte is 200 mM PBS (pH 7.0) containing 1 mM Ru(bpy)_3_^2+^ and 10 mM TPrA.

According to the finite element simulations, the TEL can reach any height from 5.3 μm to 12.1 μm by controlling *T*_e_. Therefore, optical section imaging of single cells can be achieved by image subtraction under two adjacent *T*_e_. As shown in Fig. 3b, the concentration distribution difference of Ru(bpy)_3_^2+^* between two adjacent *T*_e_ reflects a unique peak shape for the optical section at a certain height. When the image subtraction between 30 °C and 40 °C is performed, the maximal imaging layer lies 6.6 μm above the electrode surface. In this situation, the ECL section highlights cell bottom parts due to a good match of the vertical height (Fig. 3a). A shadow ECL appears at the center of cells because the upper part of cells is beyond the ECL imaging scope in this situation (30/40 °C). However, under other adjacent *T*_e_ (40/50 °C and 50/60 °C), the image subtraction makes the section move up to 9.6 μm and 13.0 μm, and thus the ECL halo gradually shrinks, reflecting the topography of the upper part of cells. It is demonstrated that cell topography is selectively imaged along the vertical direction by the subtraction of ECL images between adjacent *T*_e_. The ability of selective section imaging is also validated in other more cells. Fig. S12† shows the section imaging of another cell by the subtraction of ECL images between adjacent *T*_e_. With the increase in adjacent *T*_e_, the cell edge became less visible and the central part of the cell became brighter. The simulated lateral ECL profiles also show a brighter cell center with elevating adjacent *T*_e_ (Fig. 3c), roughly in agreement with the trend in experimental results in Fig. 3d and S12.† The deviation of lateral profiles between experimental and simulated results is probably attributed to the simulated cell shape being slightly different from the actual cell shape. The geometry module used in the COMSOL simulation shows that the cell shape is hemispheric. However, the actual cells have irregular 3D shapes from dark-field images. After overlying these cross-section images at different heights, a three-dimension cell topography is reconstructed (Fig. S13†). From the center to the edge, the cellular height gradually decreases until it approaches the electrode surface, in accord with the topography of an attached cell on a flat surface depicted by other techniques.^51^

**Fig. 4:**
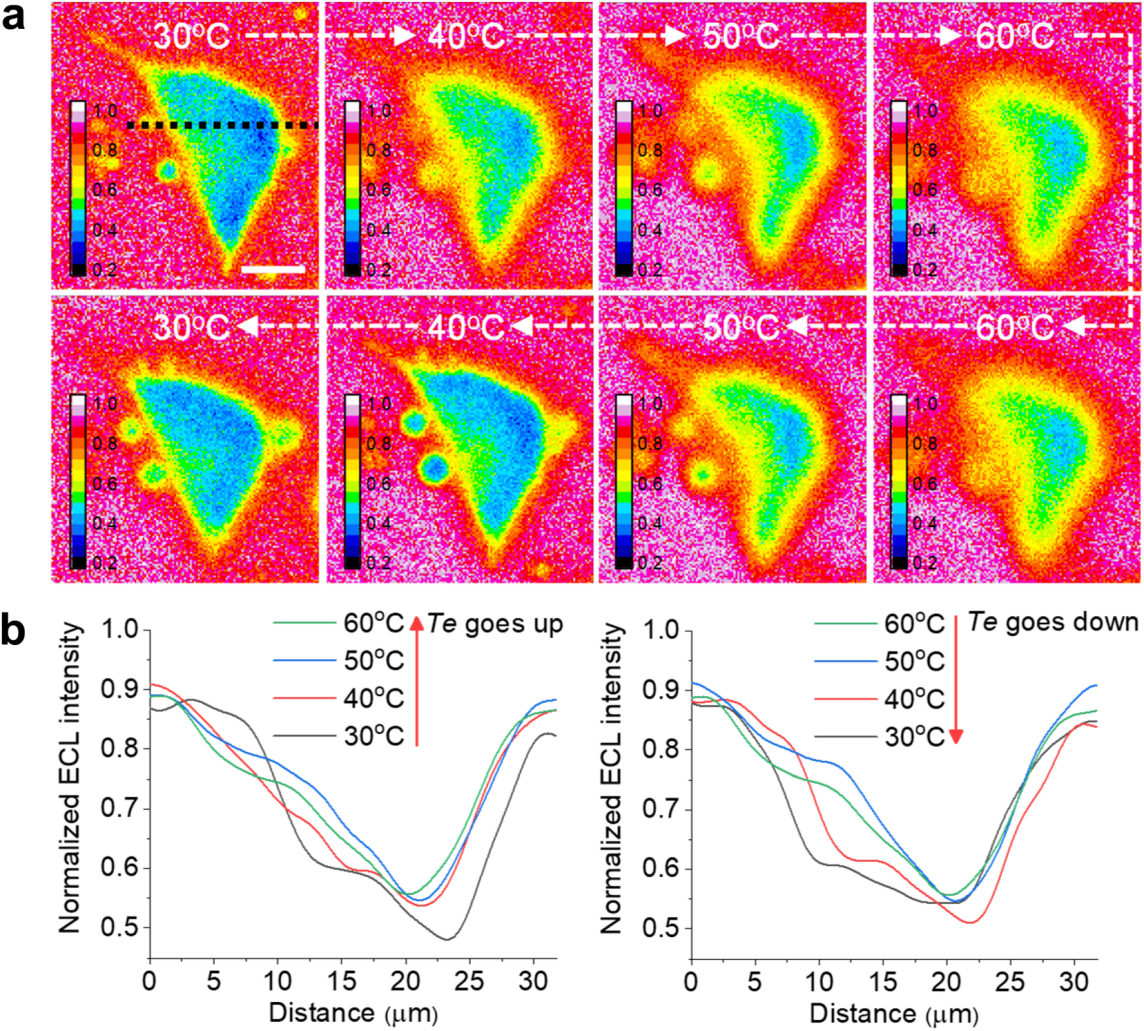
(a) Reversible ECL shadow images of an adherent cell with changing *T*_e_ between 30 °C and 60 °C. The electrolyte contains 1 mM Ru(bpy)_3_^2+^ and 10 mM TPrA in 200 mM PBS (pH = 7.0). (b) ECL profiles along the black dashed line in (a) across the cell showing the sharpness of cell edges reversibly dependent on *T*_e_ between 30 °C and 60 °C. Scale bar (white) is 10 μm.

Another advantage of the heated electrode is the reversible regulation of the ECL intensity and TEL. As shown in Fig. S14,† the peak values of both ECL intensity and anodic current are positively related to *T*_e_ during two cycles from 25 °C to 50 °C. It is attributed to the temperature dependence of the electrochemical reaction rates and diffusion rates of Ru(bpy)_3_^2+^/TPrA according to the Arrhenius equation and Stokes–Einstein equation.^7^ Subsequently, as *T*_e_ gradually decreased to 25 °C, the ECL intensity and anodic current dropped to the previous level. Similarly, the TEL also shows a reversible change as *T*_e_ goes up and down (Fig. 4a). When *T*_e_ was elevated, cellular edges became blurry, suggesting a rising TEL over the cell periphery. In contrast, as the electrode subsequently started cooling down, cell edges gradually became clear again and finally returned to the previous ECL pattern at low *T*_e_. The ECL profiles across the cell (Fig. 4b and c) indicated an opposite changing trend as the *T*_e_ went up and down. Due to the thermal expansion of the ITO electrode, the cells had to be refocused under different temperatures after *T*_e_ was stabilized. Some deviations during refocusing operation are inevitable between different temperatures. As a result, at the initial 30 °C and the latter 30 °C after a cooling process, the cell morphologies in ECL images look different. To quantify the error range, we tested more cells to record the ECL images during changing *T*_e_. As shown in Fig. S15,† the lateral ECL profiles under different temperatures showed a similar trend for all the cells. According to the statistical results (Fig. S16†), the ECL contrast in the cell central part gradually declined with elevating *T*_e_ but rebounded back with cooling down. In addition, the ECL contrast has no significant difference between the initial 30 °C image and the latter 30 °C image after a cooling process by two-tailed Student's *t*-test (*P* = 0.129). Therefore, these deviations between ECL images under the same temperature are acceptable. Thus, the heated electrode provides a flexible strategy to finely tune the ECL layer in a reversible manner.

The cell imaging based on a temperature-dependent TEL is similar to previous reports that used different ECL routes to change the TEL.^44,46^ As shown in Fig. S17,† the cellular ECL images were also regulated by the concentration of Ru(bpy)_3_^2+^ and TPrA that determined the predominant ECL reactions (oxidative reduction or catalytic route). At a high Ru(bpy)_3_^2+^/TPrA concentration ratio, the cell edges became more blurry due to the extended TEL in the catalytic route. In contrast, the low Ru(bpy)_3_^2+^/TPrA concentration ratio decreased the TEL in the oxidative reduction route so that the cell edges became clearer. The difference between the two routes is attributed to Ru(bpy)_3_^3+^ having a much longer lifetime than TPrA^+^˙ and TPrA˙. It demonstrates that ECL routes determine a base level of the TEL, but *T*_e_ provides a flexible factor to expand the regulation range around the base level. However, as a control experiment, Fig. S18† shows that 2-(dibutylamino)ethanol (DBAE) as a coreactant diminishes the change of Ru(bpy)_3_^3+^ concentration induced TEL variation. Because the DBAE radical has an even shorter lifetime than the TPrA radical, the ECL images of cell–matrix adhesions from 500 μM and 5 mM Ru(bpy)_3_^2+^ are very similar, suggesting that the change of the TEL is less obvious with the increase in Ru(bpy)_3_^3+^ concentration by using the DBAE coreactant.

**Fig. 5:**
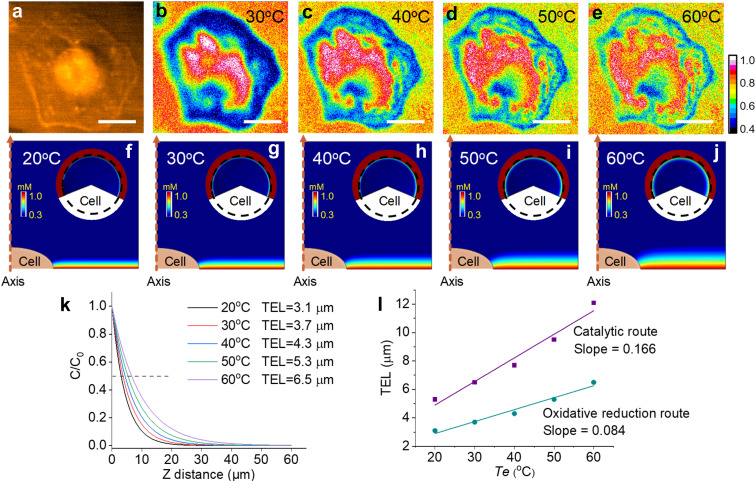
(a) Dark-field image of an adherent cell. (b–e) ECL shadow images of the cell with elevating *T*_e_ from 30 °C to 60 °C. The electrolyte contains 1 mM Ru(bpy)_3_^2+^ and 100 mM TPrA in 200 mM PBS (pH = 7.0). Scale bar (white) is 20 μm. (f–j) The side-view of the ECL layer near adherent cells at different temperatures. Inset: the top-view of ECL distribution over the adherent cell. The dashed circle indicates the outline of the cell. The primary ECL mechanism is the oxidative reduction route in 1 mM Ru(bpy)_3_^2+^ and 100 mM TPrA. (k) The normalized concentration profiles of Ru(bpy)_3_^2+^* away from the electrode surface at different temperatures. TEL is defined as the distance from the electrode surface to the site where the ECL intensity decreases to half of the maximum. (l) Comparison of TELs under the catalytic route and oxidative reduction route as a function of *T*_e_.

In the catalytic route, the diffusion distance of Ru(bpy)_3_^3+^ primarily determines the TEL, which is tuned from 5.3 μm to 12.1 μm by changing *T*_e_ from 20 °C to 60 °C. Compared with the catalytic route, the oxidative reduction route makes Ru(bpy)_3_^3+^ significantly consumed near the electrode surface by homogeneous reactions with TPrA˙ (eqn (S5)†). In this case, the temperature-dependent TEL could show a different scenario because a thinner ECL layer is anticipated. Fig. 5 shows cellular ECL images under the oxidative reduction route in 1 mM Ru(bpy)_3_^2+^ and 100 mM TPrA. The cell edges and subtle structures concealed under the cells are clearly observed at 30 °C, demonstrating a thinner ECL layer than the counterpart in the catalytic route.^48^ With elevating *T*_e_, although the contrast between cellular adhesion and blank regions is gradually weakened, these cell bottom structures are still visible even at 60 °C. It suggests that the TEL increases on a modest scale with rising temperature. To provide theoretical evidence, finite element simulation in the oxidative reduction route was performed (Fig. 5f–j). In this situation, the TEL is only 3.1 μm at 20 °C and gradually increases up to 6.5 μm at 60 °C (Fig. 5k). It is approximately half of the TEL in the catalytic route at the corresponding *T*_e_. Thus, the ECL route determined the temperature-tuned range and base level of the TEL (Fig. 5l). As shown in Fig. S19,† we recorded the ECL images under the catalytic route and oxidative reduction route in the same field of cells at the same temperature. At 30 °C, the ECL image in the oxidative reduction route clearly revealed the cell adhesion due to the thin ECL layer. In contrast, the cell contour became more blurry in the catalytic route due to the thickened ECL layer. At 50 °C, the image difference between the two ECL routes became more obvious. The ECL image under the oxidative reduction route still revealed the cell–matrix adhesion, but under the catalytic route the cell center was highlighted, indicating a thicker ECL layer in this situation. In addition, the ECL contrast as a function of voltage also reveals the difference between the two routes. In the oxidative reduction route, the ECL contrast between cellular and blank regions always monotonically rises with potential sweeping at high *T*_e_ (Fig. S20†). It demonstrates that the ECL layer is always confined near the electrode surface. But in the catalytic route, the cellular ECL contrast significantly weakens beyond 1.0 V especially at higher temperature. It indicates an obviously thickened ECL layer due to the increased diffusion distance of Ru(bpy)_3_^3+^ at high temperature.

To investigate the imaging stability of cells at high temperature, we monitored the ECL images of adherent cells at 60 °C during successive cyclic voltammetry sweeping. Fig. S21† shows that the subtle cellular structures remained unchanged as the time elapsed, demonstrating negligible damage of high *T*_e_ to the cellular morphology and affirming the stability of temperature-tuned ECL imaging.

In addition, the heated electrode can promote some electrochemical reactions which suffer from sluggish electro-kinetics at room temperature.^54^ For TPrA, its heterogeneous electrochemical oxidation is kinetically sluggish at the ITO electrode.^55^ With elevated *T*_e_, the anodic oxidation current of TPrA at 1.3 V significantly increases up to 3.4 times from 27.4 °C to 65 °C, accompanied by a lower overpotential (Fig. S22†). Similarly, with raising *T*_e_, cyclic voltammetry of Ru(bpy)_3_^2+^ shows enhanced peak currents but with a moderate degree (1.4 times) (Fig. S23†). Because the heterogeneous electron transfer kinetics of Ru(bpy)_3_^2+^ is faster than that of TPrA, the enhanced peak current of Ru(bpy)_3_^2+^ is attributed to only the accelerated diffusion rate rather than the electron transfer rate. By the synergistic effect of TPrA and Ru(bpy)_3_^2+^, the ECL intensity is significantly enhanced up to 3 times and shows a positive linear correlation with elevating *T*_e_, along with an increased oxidation current (Fig. S24†). Therefore, elevating *T*_e_ can help to improve the imaging sensitivity, especially in heterogeneous electrochemical rate-determined situations.

As shown in Fig. S25,† when the applied voltage was set to 0.96 V, the cellular adhesion showed poor contrast due to the sluggish electro-oxidation rate of TPrA and Ru(bpy)_3_^2+^ at such a low anodic potential. However, with raising *T*_e_, the ECL signal in blank regions became increasingly obvious according to temperature-dependent electrochemical reaction rates, but the insulated cellular regions did not show an increased ECL signal by the same magnitude as the blank region. Accordingly, the cellular adhesion regions were clearly illustrated at high temperature. Then we tested the ECL images at a series of anodic potentials below 1 V. All results showed a positive correlation between the *T*_e_ and ECL contrast of cell adhesion. Thus, elevating *T*_e_ facilitated the imaging sensitivity in low potential ranges.

Similarly, when low concentrations of TPrA (1 mM) and Ru(bpy)_3_^2+^ (50 μM) were used, the ECL intensity is not sufficient to distinguish cellular regions due to weak ECL signals (Fig. S26†). But elevated *T*_e_ accelerated electro-kinetics and diffusion rates, improving the ECL intensity in blank regions. Thus, the cellular contrast was intensified (from 6% to 16%) with rising temperature and the cellular adhesion became distinguishable. In addition, the regulation of cellular contrast showed a reversible characteristic as *T*_e_ went up and down (Fig. S27†). With the *T*_e_ decreasing from 60 °C to 30 °C, the ECL contrast of cell regions gradually declined to the previous level, following the opposite trajectory to the ECL contrast in the *T*_e_ increasing process. It demonstrates that *T*_e_ is a flexible parameter to regulate the imaging contrast.

## Conclusions

A temperature-regulated ECL imaging analysis of cells is demonstrated by combining a heated electrode and optical microscope. The TEL in the catalytic route and oxidative route shows different regulation ranges as a function of *T*_e_. At a high concentration of Ru(bpy)_3_^2+^, the catalytic route depending on the diffusion of Ru(bpy)_3_^3+^ allows an obvious extension of the TEL from the cell bottom to the top. Therefore, the section imaging at different heights of the cell is enabled by subtracting images at adjacent *T*_e_. On the other hand, the ECL layer in the oxidative reduction route is confined to the electrode surface and shows a moderate extension with elevated *T*_e_. Thus, the temperature-tuned ECL layer provides two kinds of imaging scope for adherent cells. In addition, elevating *T*_e_ promotes sluggish electro-kinetics and enhances the image sensitivity of cell adhesion in heterogeneous electrochemical rate-determined situations. Because temperature is a key parameter in electrochemical processes, altering *T*_e_ offers a flexible and reversible approach to regulate the TEL and contrast for ECL imaging. Also, the relationship between temperature and ECL scenarios can in turn help to design a thermal imaging technique for heat generation from biological processes.

## Experimental section

### Cell culture and fixing on the ITO electrode

HeLa cells were cultured in DMEM containing 10% fetal calf serum, penicillin (80 U mL^−1^), and streptomycin (0.08 mg mL^−1^) in an incubator (ThermoFlash Scientific) at 37 °C under an atmosphere with 5% CO_2_ and 90% relative humidity. When the cells were at 80% confluence, they were separated from the cell culture medium and washed twice with PBS, and then were digested for 2 min by trypsin (0.25%, m/v). After centrifuging at 1200 rpm for 5 min to gather the cells, the cells were resuspended in 1 mL of DMEM for seeding on an electrode. Firstly, ITO glass was successively cleaned in an ultrasonic bath with ultrapure water, ethanol, and ultrapure water for 3 min, and was then dried with a nitrogen flow. Next, 500 μL DMEM and 80 μL of prepared cell resuspension were added onto the ITO surface with an area of 1.5 × 1.5 cm^2^, and then cultured in an incubator overnight. Before measurement, the electrode with cells was washed with PBS twice, and fixed by 4% paraformaldehyde solution for 15 min, and then washed with PBS twice.

### ECL intensity measurements with PMT

The ECL emission measurements were conducted on a model MPI-E ECL analyzer (Xi'an Remex Analytical Instrument Co., Ltd, China) with a three-electrode system including a working electrode (ITO, surface resistivity < 10 Ω per square, thickness 1800 ± 300 Å, Zhongjingkeyi Technology Co., Ltd), a Pt counter electrode, and an Ag/AgCl (saturated KCl) reference electrode. The measurements of ECL intensity at different temperatures were performed with a high voltage of −600 V. At first, an ITO electrode was fixed on a heating plate, and both of them were placed in a detection box. Next, the prepared solutions were added on the surface of ITO, and then ECL was triggered by cyclic voltammetry scan between 0.0 V and 1.3 V with a rate of 0.1 V s^−1^. The emission was collected by the PMT simultaneously. It should be pointed out that before changing the temperature, the system was pretreated with some cycles of cyclic voltammetry scan until the ECL intensity was stable at room temperature, and then different temperature was set for further measurements.

### Imaging measurements

The ECL imaging setup included two major parts. An electron multiplying CCD (EMCCD) (Evolve delta, Photometrics, Scientifica, U.S.A) was used to collect signals and output images. A CHI660D electrochemical workstation (CH Instruments Co., China) was used to generate and record electrochemical responses. The optical path system was composed of a water immersion objective (60×, NA 1.1, Olympus, Japan) and a tube lens. The ECL imaging test must be operated in a dark box. A traditional three-electrode system was used for all electrochemical experiments, which consisted of ITO glass as the working electrode, a platinum wire as the auxiliary electrode, and Ag/AgCl (saturated KCl) as the reference electrode. All imaging measurements were performed with the above mentioned ECL imaging instrument. Unless otherwise stated, ECL was triggered by cyclic voltammetry scan between 0.0 V and 1.3 V with a rate of 0.1 V s^−1^, and the emission was collected by an EMCCD with 0.2 s exposure time and 50 EM Gain. As for temperature related experiments, an ITO electrode was initially placed on the heating plate, and then different temperatures were set for further measurements. The ITO electrode surface was refocused under different setting temperatures to eliminate the out-of-focus effect from thermal expansion of ITO. The digital temperature controller and the corresponding heating plate were purchased from Jiukou hardware store (China) and used in all temperature related experiments.

## Data availability

The data and computer codes supporting the findings of this study are available from the authors upon reasonable request.

## Author contributions

C. M., Z. X., L. P. J. and J. J. Z. conceived the study. Z. X. and C. M. performed the experiments. Z. X., C. M. and X. G. built the microscope and analyzed the data. C. M., L. P. J. and J. J. Z. advised on the manuscript. C. M., Z. X. and J. J. Z. wrote the paper.

## Conflicts of interest

The authors declare no competing financial interest.

## Supplementary Material

SC-013-D2SC04944A-s001
