# Supplementary material for: A temperature-tuned electrochemiluminescence layer for reversibly imaging cell topography
Source: Chem Sci. 2022 Nov 10;13(46):13938–47. doi: 10.1039/d2sc04944a (PMC9710227; doi:10.1039/d2sc04944a)
Supplement: SC-013-D2SC04944A-s001 [file SC-013-D2SC04944A-s001.pdf]

Electronic Supplementary Material (ESI) for Chemical Science.
This journal is © The Royal Society of Chemistry 2022

Supporting Information

Temperature-Tuned Electrochemiluminescence Layer for Reversibly Imaging Cell Topography

Cheng Ma,[+] Zejing Xing,[+] Xiaodan Gou, Li-Ping Jiang* and Jun-Jie Zhu*

Abstract: Investigating electrochemiluminescence (ECL) scenario under different temperatures is important to expand its imaging scope near electrode surface. Here, we discover a temperature-tuned ECL layer by recording the evolution of shadow regions of adherent cells. Finite element simulation and experimental results demonstrate that the thickness of ECL layer (TEL) is reversibly regulated by electrode temperature (*Te*) so that single cell topography at different heights is imaged. The TEL in two ECL routes shows different regulation ranges with elevated *Te*, thus providing a flexible approach to adjust imaging scope within specific heights. In addition, heated electrode significantly improves the image quality of cell adhesion in heterogeneous electrochemical rate-determined situations. Thus, the contrast in cell regions shows a reversible response to *Te*. This work provides a new approach to regulate TEL and is promising for monitoring transient heat generation from biological entities.

Table of Contents

Results and Discussion 3
Experimental section 16
References 16
Author Contributions 16

Results and Discussion

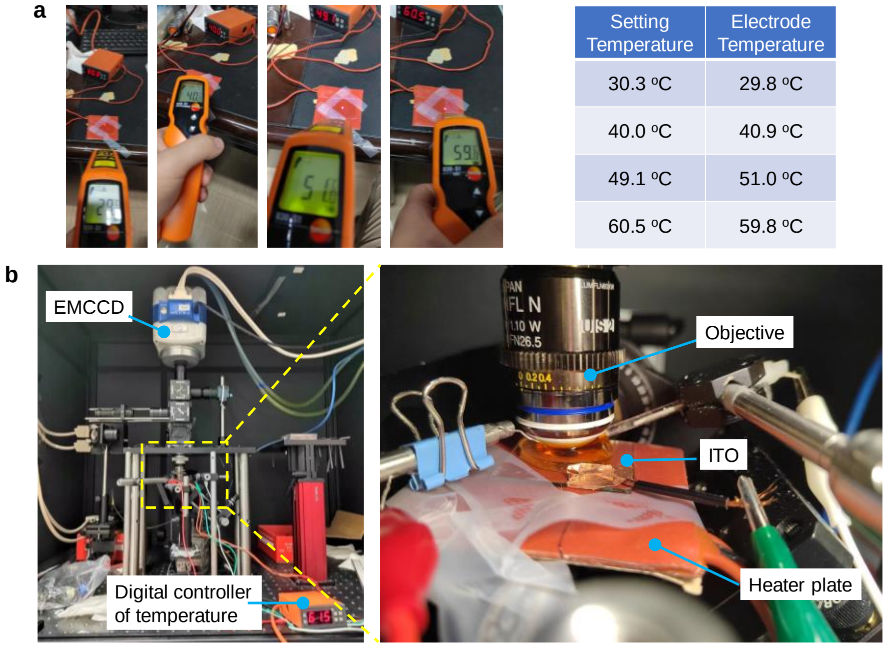

Setting Temperature	Electrode Temperature
30.3 ºC	29.8 ºC
40.0 ºC	40.9 ºC
49.1 ºC	51.0 ºC
60.5 ºC	59.8 ºC

Figure S1. (a) Electrode temperature measured by infrared thermometer under different setting temperatures of thermostat. (b) Photograph of heating setup and upright microscope (left panel). The zoom-in picture showing the configuration of heater plate, ITO, and objective (right panel).

An infrared thermometer is used to measure the electrode temperature, for infrared ray does not penetrate ITO glass. Because ITO electrode is very thin (1.1 mm thickness), the temperature on ITO electrode surface is approximately equal to that on the heater plate (Figure S1). The deviation between the two is no more than 2ºC when setting temperature ranges from 30ºC to 60ºC.

Catalytic route:
$Ru(bpy)_3^{2+} - e^- = Ru(bpy)_3^{3+}$ (heterogeneous electron transfer)
$Ru(bpy)_3^{3+} + TPrA = Ru(bpy)_3^{2+} + TPrA^{\bullet+}$ (homogeneous reactions)
$TPrA^{\bullet+} - H^+ = TPrA^{\bullet}$ (homogeneous reactions)
$Ru(bpy)_3^{3+} + TPrA^{\bullet} = Ru(bpy)_3^{2+*} + P_1$ (homogeneous reactions)
$Ru(bpy)_3^{2+*} = Ru(bpy)_3^{2+} + h\nu$ (ECL emission)

Oxidative reduction route:
$Ru(bpy)_3^{2+} - e^- = Ru(bpy)_3^{3+}$ (heterogeneous electron transfer)
$TPrA - e^- = TPrA^{\bullet+}$ (heterogeneous electron transfer)
$TPrA^{\bullet+} - H^+ = TPrA^{\bullet}$ (homogeneous reactions)
$Ru(bpy)_3^{3+} + TPrA^{\bullet} = Ru(bpy)_3^{2+*} + P_1$ (homogeneous reactions)
$Ru(bpy)_3^{2+*} = Ru(bpy)_3^{2+} + h\nu$ (ECL emission)

Low oxidative potential route:
$TPrA - e^- = TPrA^{\bullet+}$ (heterogeneous electron transfer)
$TPrA^{\bullet+} - H^+ = TPrA^{\bullet}$ (homogeneous reactions)
$Ru(bpy)_3^{2+} + TPrA^{\bullet} = Ru(bpy)_3^{+} + P_1$ (homogeneous reactions)
$Ru(bpy)_3^{+} + TPrA^{\bullet+} = Ru(bpy)_3^{2+*} + TPrA$ (homogeneous reactions)
$Ru(bpy)_3^{2+*} = Ru(bpy)_3^{2+} + h\nu$ (ECL emission)

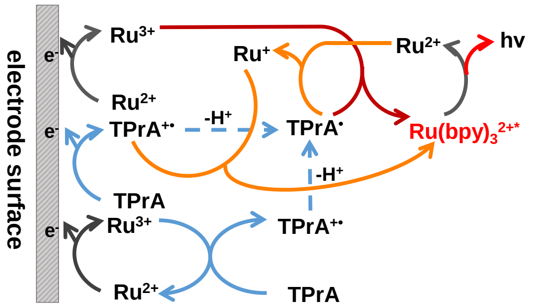

Scheme S1. The ECL reaction steps in "catalytic route", "oxidative reduction route" and "low oxidation potential route" in $Ru(bpy)_3^{2+}$ and tripropylamine (TPrA) solution.[1] In high concentration of $Ru(bpy)_3^{2+}$, catalytic route is prevailing. Otherwise, oxidative reduction route and low oxidation potential route will be dominant in low concentration of $Ru(bpy)_3^{2+}$. $TPrA^{\bullet+}$ is $Pr_3N^{\bullet+}$, $TPrA^{\bullet}$ is $Pr_2NC^{\bullet}HCH_2CH_3$, P_1 is $Pr_2N^+{=}CHCH_2CH_3$.

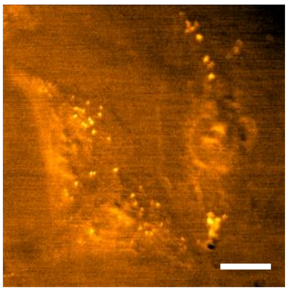

Figure S2. Dark-field image of the three cells in Figure 2. An oblique illumination highlights the height of different subcellular regions. Scale bar is 20 μm.

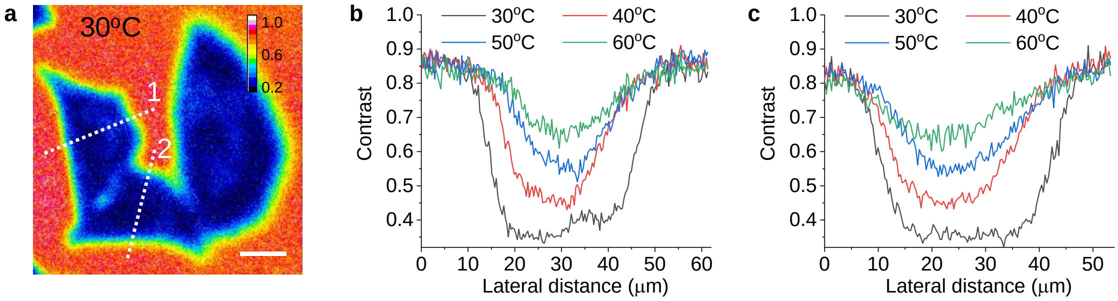

Figure S3. ECL intensity profiles across the other two cells in Figure 2b at different *Te*. (a) Cellular shadow ECL pattern at 30°C. (b,c) Normalized ECL intensity profiles along the line 1 (b) and line 2 (c) in (a) with elevated *Te*.

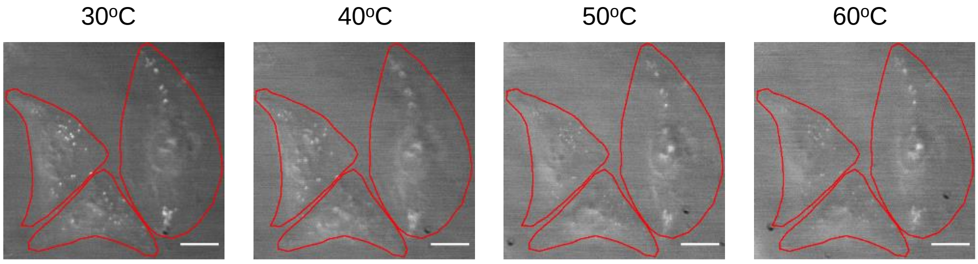

Figure S4. Dark-field images of cells with elevating electrode temperature from 30°C to 60°C. Scale bar is 20 μm.

COMSOL simulation

COMSOL Multiphysics (Version 5.6) was used for digital simulations. Because the mechanisms for ECL generation are so complex (Scheme S1), only the main routes are considered, which involved "oxidative reduction route" and "catalytic route". The reaction equations are as follows,

$$TPrAH^{+} \xrightarrow{k_1} TPrA + H^{+} \quad (S1)$$

$$TPrA \xrightarrow{k_2} TPrA^{\bullet +} + e^{-} \quad (S2)$$

$$TPrA^{\bullet +} \xrightarrow{k_3} TPrA^{\bullet} + H^{+} \quad (S3)$$

$Ru(bpy)_3^{2+} \xrightarrow{k_4} Ru(bpy)_3^{3+} + e^-$ (S4)

$Ru(bpy)_3^{3+} + TPrA^{\bullet} \xrightarrow{k_5} Ru(bpy)_3^{2+*} + P1$ (S5)

$Ru(bpy)_3^{3+} + TPrA \xrightarrow{k_6} Ru(bpy)_3^{2+} + TPrA^{\bullet +}$ (S6)

$Ru(bpy)_3^{2+*} \xrightarrow{k_7} Ru(bpy)_3^{2+} + hv$ (S7)

$TPrA^{\bullet} \xrightarrow{k_8} P1 + e^-$ (S8)

Table S1. Reaction rates in the ECL processes.

Name	Value	Description
k1	8 1/s	Rate constant of eq. S1
k2	10^{-4} m/s	Rate constant of eq. S2
k3	3500 1/s	Rate constant of eq. S3
k4	0.15 m/s	Rate constant of eq. S4
k5	10^6 $m^3/(s*mol)$	Rate constant of eq. S5
k6	13 $m^3/(s*mol)$	Rate constant of eq. S6
k7	300 1/s	Rate constant of eq. S7
k8	10^{-4} m/s	Rate constant of eq. S8

All the reaction rate constants are listed in Table S1. These reactions can be divided into two groups: heterogeneous reactions involving reactions S2, S4, S8 occur on electrode surface and other reactions occur in the solution are homogeneous reactions. The parameters involving electrochemical reaction rate constants and diffusion coefficients will change at different temperatures while others are constant. Although high temperature facilitated electrochemical reaction rates according to Arrhenius equation (eq. S9), simulation results showed the variety of the thickness of ECL-emitting layer was negligible with different electrochemical reaction rate constants at diffusion-controlled voltage ranges.

$$k = Ae^{-\frac{E_a}{RT}} \quad \text{(S9)}$$

k is the reaction rate constant, A is Arrhenius constant, E_a is the activation energy, T is the absolute temperature. R is the molar gas constant. Instead, the thickness of ECL layer would extend obviously when diffusion coefficients increased. Thus varied diffusion behaviors caused by different temperatures are the main reason for the changed thickness of ECL layer. From the 2D axisymmetric geometry as shown in Figure S5, we assumed that the diffusion behavior was only in the vertical direction by setting the upper boundary at the initial concentration and no flux at the right boundary.

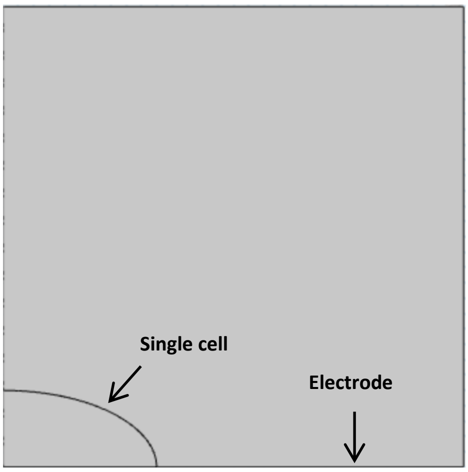

Figure S5. The geometry module used in the COMSOL simulation

Given the known diffusion coefficient of $Ru(bpy)_3^{2+}$ ($5.9*10^{-9}$ m^2/s) at room temperature, the diffusion coefficients of $Ru(bpy)_3^{2+}$ with elevating Te were calculated by Randles-Sevcik equation (eq. S10 and Figure S6).

$$i_p = 0.4463(\frac{F^3}{RT})^{1/2} n^{3/2} A D_0^{1/2} C_0^* v^{1/2} \quad \text{(S10)}$$

where i_p is the peak current, D_0 is the diffusion coefficient, n is the number of transferred electrons in $Ru(bpy)_3^{2+}$ oxidation, A is the area of electrode surface, T is absolute temperature, R is the molar gas constant, C_0^* is the bulk concentration of $Ru(bpy)_3^{2+}$, F is Faraday constant, υ is scan rate.

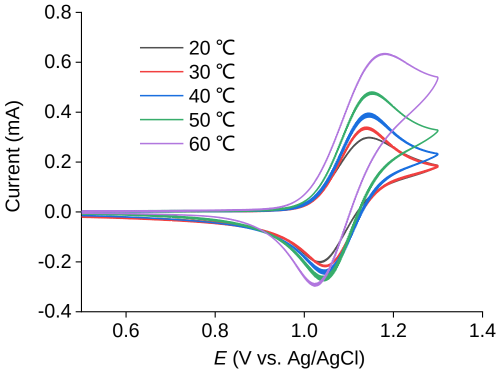

Figure S6. Cyclic voltammetry (CV) of 1 mM $Ru(bpy)_3^{2+}$ with elevated *Te*. Scan rate is 0.1 V/s. According to i_p values from CV curves, the diffusion coefficients of $Ru(bpy)_3^{2+}$ at different temperatures are calculated by Randles-Sevcik equation.

In the vicinity of electrode, when the temperature of electrode surface increased, the viscosity of the solution there would decrease, causing a larger diffusion coefficient according to Stokes-Einstein equation (eq. S11).

$$D = kT/6\pi r_i \eta \qquad \text{(S11)}$$

where D is the diffusion coefficient, k is Boltzmann's constant, T is the absolute temperature, r_i is the radius of the diffusing species, and η is the viscosity of the solution. Given the known diffusion coefficients of TPrA, H^+ and Buf involved in ECL reactions at room temperature, their diffusion coefficients will increase as the same magnitude as $Ru(bpy)_3^{2+}$ diffusion coefficient in the solution when the temperature increased. Because the heating process is slow and *Te* usually maintain more than 5 min before measuring ECL, convection between electrode and bulk solution is not significant. So the heating method is similar to isothermal heating method. The diffusion coefficients of all the species at different temperatures are listed in Table S2. The initial concentrations of all species were set the same as experiment condition and listed in Table S3.

Table S2. Diffusion coefficient of all species at different temperatures.

Temperature	Name	Value	Description
20°C	DTPrA	$5*10^{-9}$ m²/s	Diffusion coefficient of TPrA and its derivative at 20°C
	DRu	$5.9*10^{-9}$ m²/s	Diffusion coefficient of $Ru(bpy)_3^{2+}$ and its derivative at 20°C
	DH	$5*10^{-9}$ m²/s	Diffusion coefficient of H^+ at 20°C
	DBuf	$5*10^{-10}$ m²/s	Diffusion coefficient of buffer at 20°C
30°C	DTPrA	$7.17*10^{-9}$ m²/s	Diffusion coefficient of TPrA and its derivative at 30°C
	DRu	$8.47*10^{-9}$ m²/s	Diffusion coefficient of $Ru(bpy)_3^{2+}$ and its derivative at 30°C
	DH	$7.17*10^{-9}$ m²/s	Diffusion coefficient of H^+ at 30°C
	DBuf	$7.17*10^{-10}$ m²/s	Diffusion coefficient of buffer at 30°C
40°C	DTPrA	$1.02*10^{-8}$ m²/s	Diffusion coefficient of TPrA and its derivative at 40°C
	DRu	$1.20*10^{-8}$ m²/s	Diffusion coefficient of $Ru(bpy)_3^{2+}$ and its derivative at 40°C
	DH	$1.02*10^{-8}$ m²/s	Diffusion coefficient of H^+ at 40°C
	DBuf	$1.02*10^{-9}$ m²/s	Diffusion coefficient of buffer at 40°C
50°C	DTPrA	$1.50*10^{-8}$ m²/s	Diffusion coefficient of TPrA and its derivative at 50°C
	DRu	$1.77*10^{-8}$ m²/s	Diffusion coefficient of $Ru(bpy)_3^{2+}$ and its derivative at 50°C
	DH	$1.50*10^{-8}$ m²/s	Diffusion coefficient of H^+ at 50°C
	DBuf	$1.50*10^{-9}$ m²/s	Diffusion coefficient of buffer at 50°C
60°C	DTPrA	$2.29*10^{-8}$ m²/s	Diffusion coefficient of TPrA and its derivative at 60°C
	DRu	$2.70*10^{-8}$ m²/s	Diffusion coefficient of $Ru(bpy)_3^{2+}$ and its derivative at 60°C
	DH	$2.29*10^{-8}$ m²/s	Diffusion coefficient of H^+ at 60°C
	DBuf	$2.29*10^{-9}$ m²/s	Diffusion coefficient of buffer at 60°C

Table S3. Initial concentration of all species.

Name	Value	Description
cTPrA0	10 mol/m^3 or 100 mol/m^3	Initial concentration of TPrA
cRu02	1 mol/m^3	Initial concentration of $Ru(bpy)_3^{2+}$
cH0	3.98*10^{-5} mol/m^3	Initial concentration of H^+
cBuf0	200 mol/m^3	Initial concentration of buffer

In addition, all redox species were assumed that they transport exclusively by diffusion, thus the transition conditions follow the Fick's second law.

$$\frac{\partial C_i}{\partial t} = \mathrm{D_i}\nabla^2 C_i \qquad \text{(S12)}$$

As results, the concentration change of all species in the above reactions can be described in Table S4. Some parameters in this simulation such as rate constants and diffusion coefficients are referred to previous works.[2-4]

Table S4. The concentration changes of all species with time.

The concentration change of TPrA	$\frac{\partial[\mathrm{TPrA}]}{\partial t} = D_{\mathrm{TPrA}}\Delta[\mathrm{TPrA}] - k_6[\mathrm{Ru}^{3+}][\mathrm{TPrA}]$
The concentration change of $TPrA^{\bullet+}$	$\frac{\partial[\mathrm{TPrA}^{\bullet+}]}{\partial t} = D_{\mathrm{TPrA}^{\bullet+}}\Delta[\mathrm{TPrA}^{\bullet+}] - k_3[\mathrm{TPrA}^{\bullet+}] + k_6[\mathrm{TPrA}][\mathrm{Ru}^{3+}]$
The concentration change of $TPrA^{\bullet}$	$\frac{\partial[\mathrm{TPrA}^{\bullet}]}{\partial t} = D_{\mathrm{TPrA}^{\bullet}}\Delta[\mathrm{TPrA}^{\bullet}] + k_3[\mathrm{TPrA}^{\bullet+}] - k_5[\mathrm{Ru}^{3+}][\mathrm{TPrA}^{\bullet}]$
The concentration change of Ru^{2+}	$\frac{\partial[\mathrm{Ru}^{2+}]}{\partial t} = D_{\mathrm{Ru}^{2+}}\Delta[\mathrm{Ru}^{2+}] + k_6[\mathrm{Ru}^{3+}][\mathrm{TPrA}] + k_7[\mathrm{Ru}^{2+*}]$
The concentration change of Ru^{3+}	$\frac{\partial[\mathrm{Ru}^{3+}]}{\partial t} = D_{\mathrm{Ru}^{3+}}\Delta[\mathrm{Ru}^{3+}] - k_5[\mathrm{Ru}^{3+}][\mathrm{TPrA}^{\bullet}] - k_6[\mathrm{Ru}^{3+}][\mathrm{TPrA}]$
The concentration change of Ru^{2+}*	$\frac{\partial[\mathrm{Ru}^{2+*}]}{\partial t} = k_5[\mathrm{Ru}^{3+}][\mathrm{TPrA}^{\bullet}] - k_7[\mathrm{Ru}^{2+*}]$

In this simulation, for simplicity we conducted it at a single cell and constructed a 2D axisymmetric geometry (Figure S5). "Transport of Diluted Species" physical field was utilized to study the time-dependent transport of diluted species in ECL reactions. It must also be mentioned that because the reactions at electrode surface are fast enough and almost be diffusion-controlled, the flux (J) boundaries at electrode surface can be expressed as

$$\mathrm{J}_{TPrA} = -\mathrm{J}_{TPrA^{\bullet+}} = -k_2[TPrA] \qquad \text{(S13)}$$

$$\mathrm{J}_{Ru^{2+}} = -\mathrm{J}_{Ru^{3+}} = -k_4[Ru^{2+}] \qquad \text{(S14)}$$

$$\mathrm{J}_{TPrA^{\bullet}} = -\mathrm{J}_{P1} = -k_8[TPrA^{\bullet}] \qquad \text{(S15)}$$

where $[TPrA]$, $[Ru^{2+}]$ and $[TPrA^{\bullet}]$ are the concentration of $TPrA$, $\mathrm{Ru(bpy)}_3^{2+}$ and $TPrA^{\bullet}$, respectively.

The mesh setting is presented in Figure S7. Because the geometry of the module was simple, the mesh setting was extremely fine.

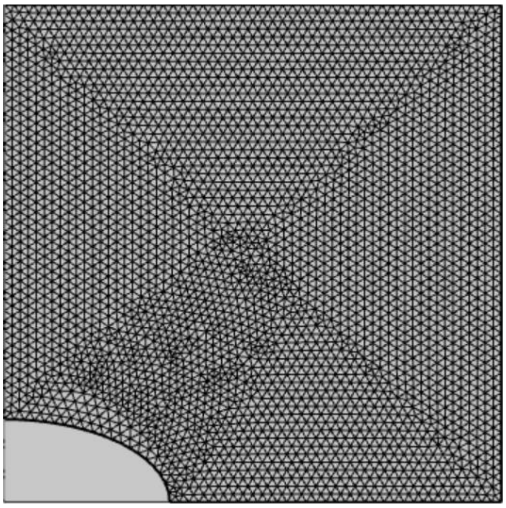

Figure S7. Mesh setting of a single cell in COMSOL software.

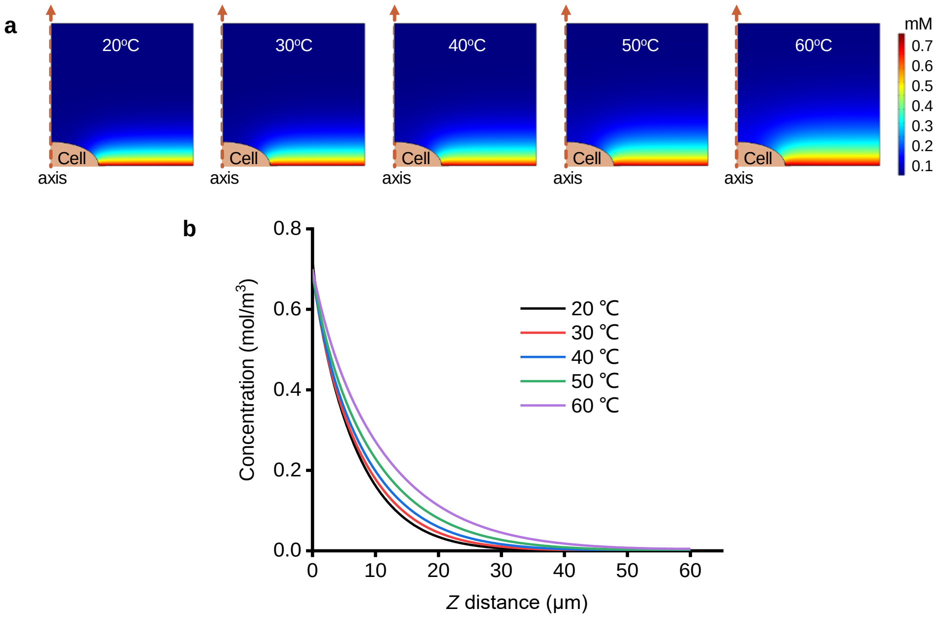

Figure S8. (a) The side-view of steady state concentration pattern of $Ru(bpy)_3^{3+}$ at different temperatures. (b) The concentration profiles of $Ru(bpy)_3^{3+}$ away from electrode surface at different temperatures.

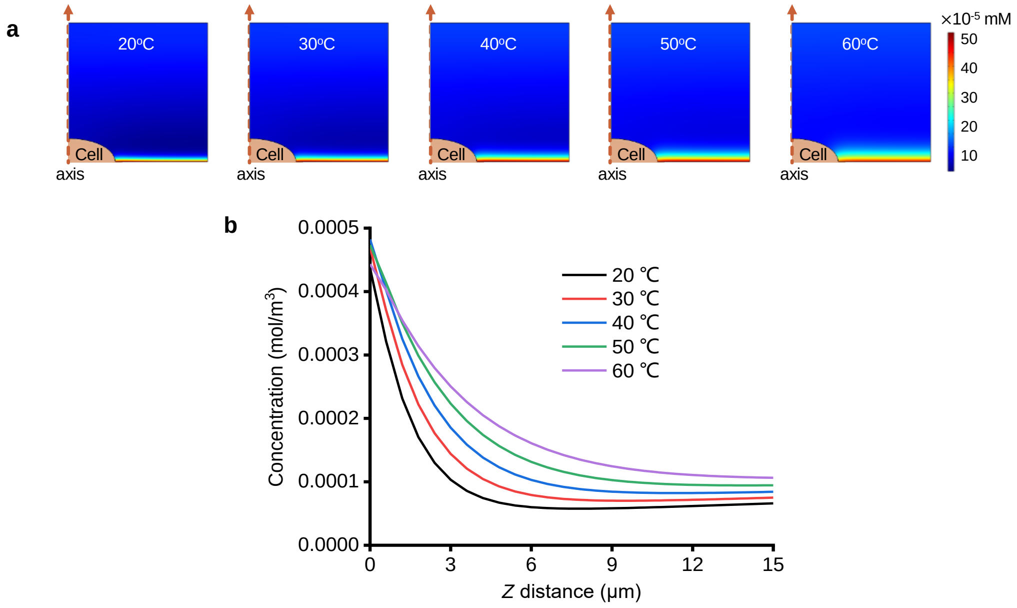

Figure S9. (a) The side-view of steady state concentration pattern of TPrA$^{\bullet}$ at different temperatures. (b) The concentration profiles of TPrA$^{\bullet}$ away from electrode surface at different temperatures.

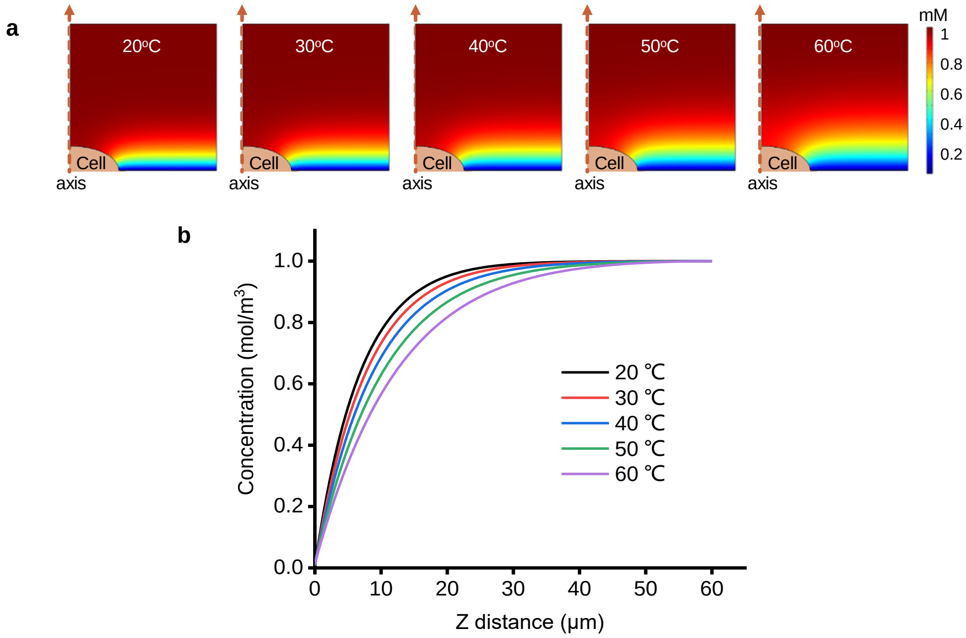

Figure S10. (a) The side-view of steady state concentration pattern of $Ru(bpy)_3^{2+}$ at different temperatures. (b) The concentration profiles of $Ru(bpy)_3^{2+}$ away from electrode surface at different temperatures.

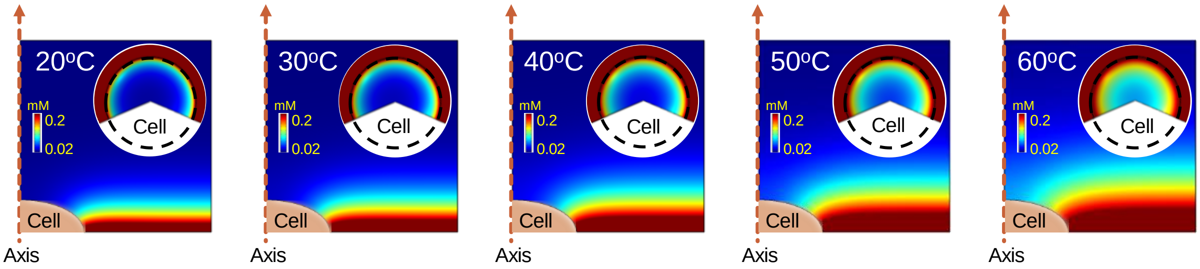

Figure S11. The side-view of ECL layer near an adherent cell at different temperatures. Inset: the top-view of ECL distribution over an adherent cell. The dash circle denotes the outline of cell. The primary ECL mechanism is catalytic route in 1 mM $Ru(bpy)_3^{2+}$ and 10 mM TPrA.

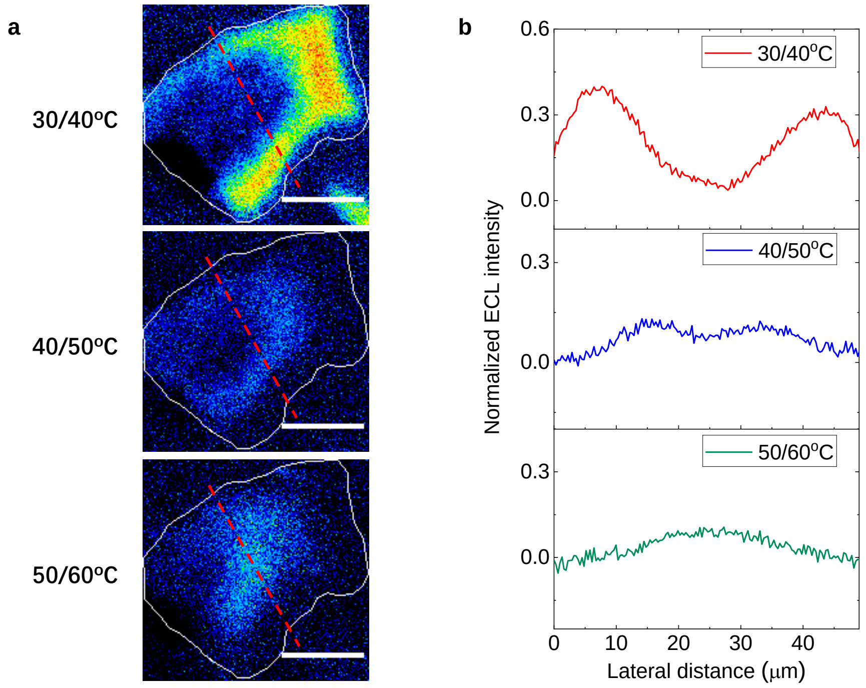

Figure S12. (a) ECL section of cells by image subtraction under two adjacent *Te*. The white lines denote the cellular edges. Scale bar (white) is 20 μm. (b) Experimental ECL profiles along the red dash lines in (a). Electrolyte is 200 mM PBS (pH 7.0) containing 1 mM $Ru(bpy)_3^{2+}$ and 10 mM TPrA.

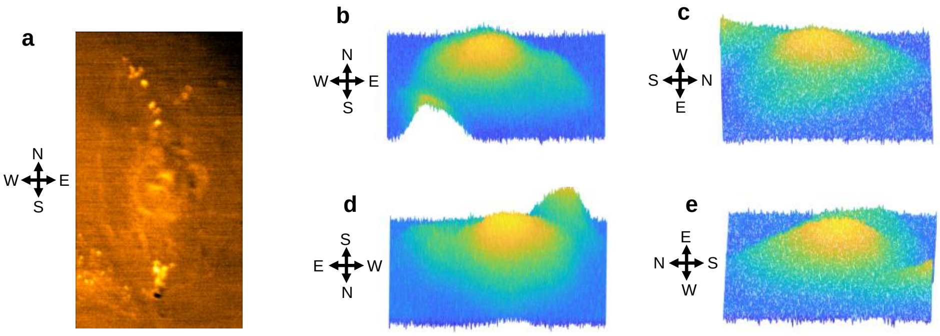

Figure S13. (a) Dark-field image of an adherent cell. (b-e) Three-dimensional topography of the cell in (a) from four different perspectives after the reconstruction of optical section ECL images of the single cell in Figure 3a.

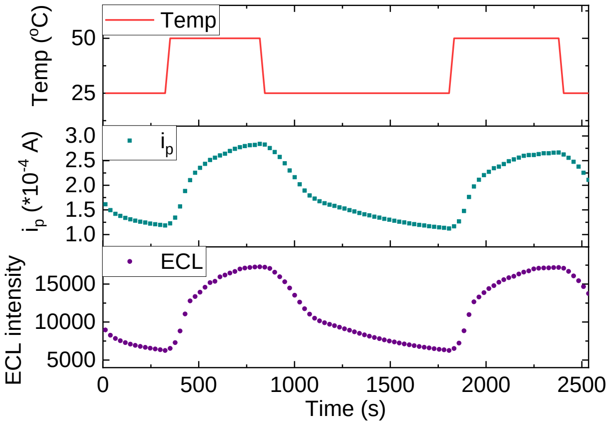

Figure S14. The trajectories of anodic current peak and ECL peak with changing electrode temperature, showing a reversible response as a function of electrode temperature. Continuous cyclic voltammetry scan from 0 V to 1.3 V was employed as the electrode temperature goes up and down. Electrolyte is 200 mM PBS (pH 7.0) containing 2 mM $Ru(bpy)_3^{2+}$and 5 mM TPrA.

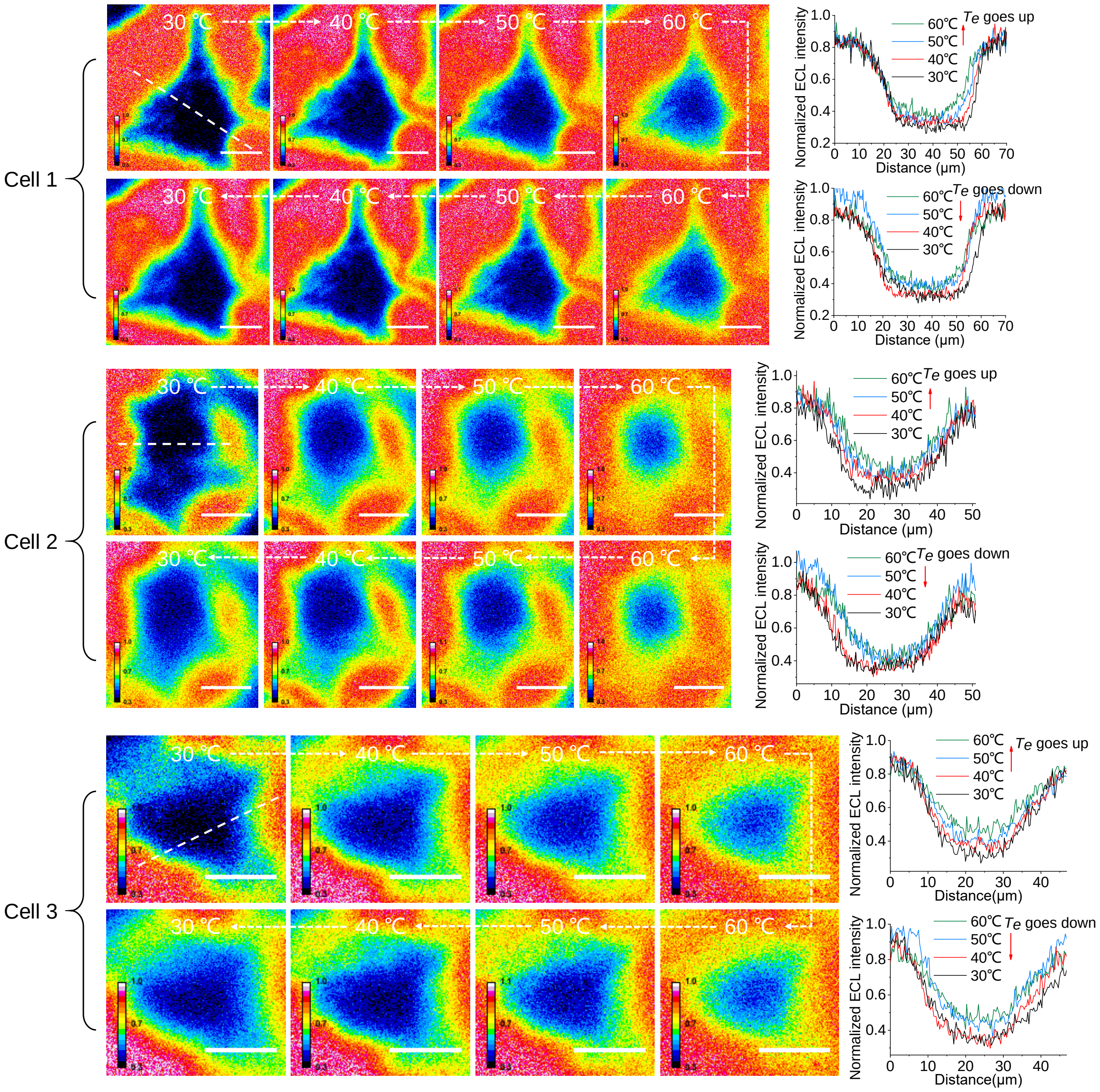

Figure S15. Reversible ECL shadow images of three adherent cells with changing *Te* between 30ºC and 60ºC. The right panel shows ECL profiles along the white dash line across the cell showing the sharpness of cell edges reversibly dependent on *Te* between 30ºC and 60ºC. The electrolyte contains 1 mM $Ru(bpy)_3^{2+}$ and 10 mM TPrA in 200 mM PBS (pH = 7.0). Scale bar (white) is 20 μm.

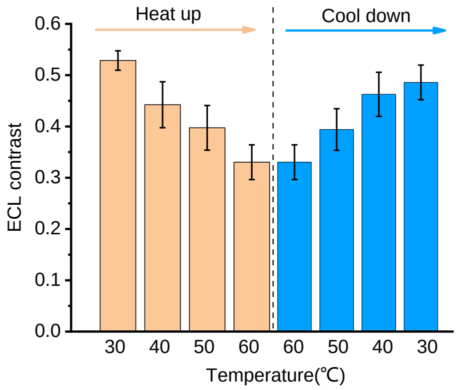

Figure S16. ECL contrast of central part of the three cells in Figure S15, showing a good reversibility with changing *Te* between 30ºC and 60ºC. The ECL contrast has no significant difference between the two 30ºC by two-tailed Student's t-test (P = 0.129), as well as between other same temperature.

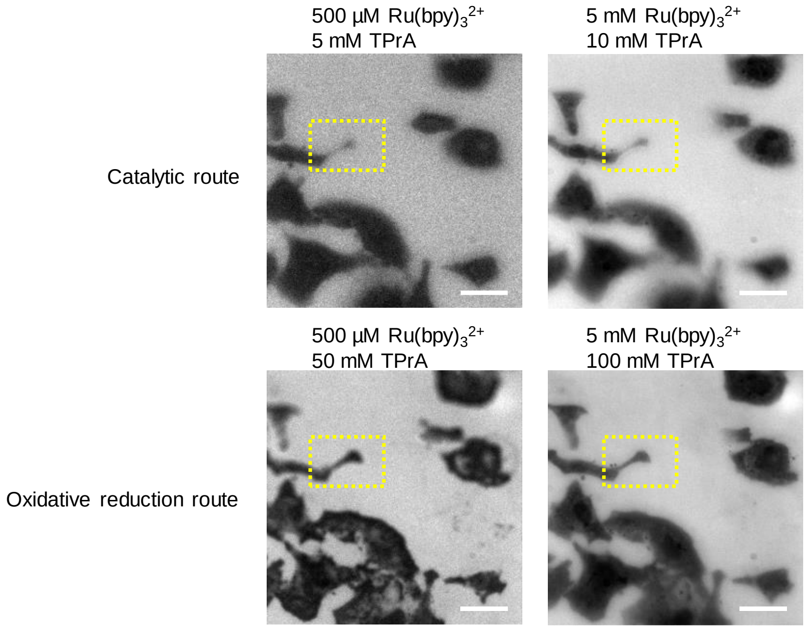

Figure S17. ECL images of adherent cells under catalytic route and oxidative reduction route, respectively. In oxidative reduction route, the TPrA concentration is 10 times higher than that in catalytic route. The yellow boxes indicate cell-matrix adhesion. Scale bar (white) is 30 μm.

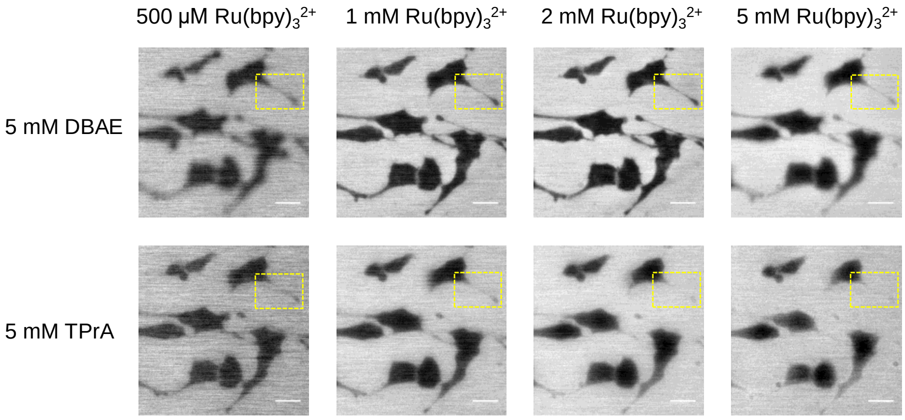

Figure S18. ECL images of adherent cells using 5 mM DBAE or TPrA as coreactant with the increasing concentration of $Ru(bpy)_3^{2+}$ from 500 μM to 5 mM. The yellow box indicates ECL pattern of a cell adhesion region at different condition. Scale bar (white) is 20 μm.

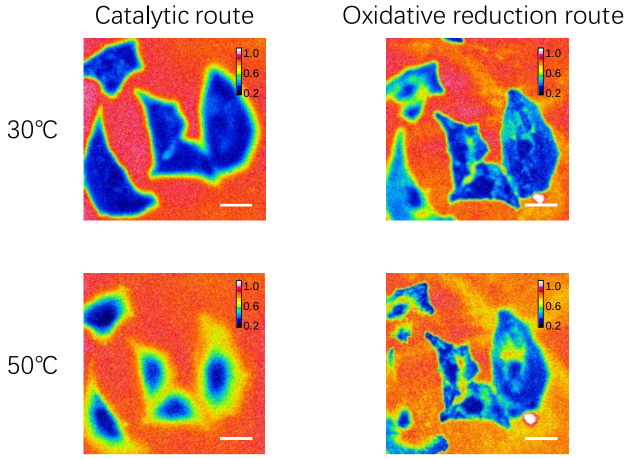

Figure S19. ECL images under the catalytic route and oxidative reduction route in the same field of cells at same 30ºC and 50ºC. In catalytic route, the electrolyte contains 1 mM $Ru(bpy)_3^{2+}$ and 10 mM TPrA in 200 mM PBS (pH = 7.0). In oxidative reduction route, the electrolyte contains 1 mM $Ru(bpy)_3^{2+}$ and 100 mM TPrA in 200 mM PBS (pH = 7.0). The TPrA concentration is 10 times higher than that in catalytic route. Scale bar (white) is 20 µm.

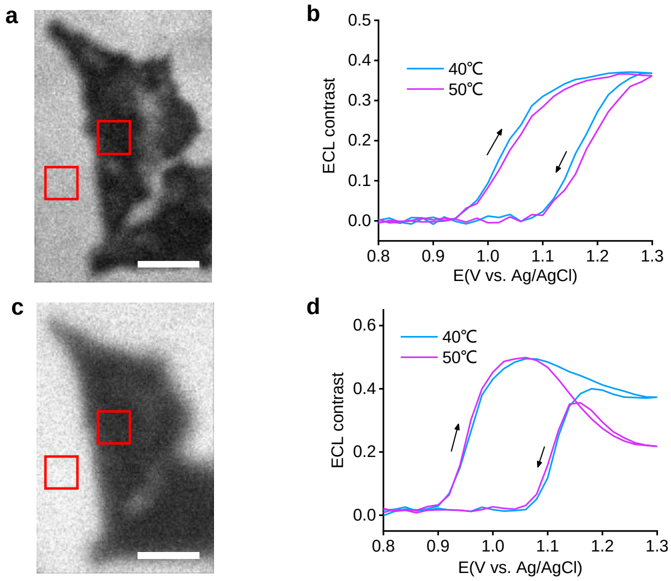

Figure S20. (a) ECL shadow image of cells with oxidative reduction route in 1 mM $Ru(bpy)_3^{2+}$ and 100 mM TPrA. (b) The profiles of ECL contrast between cell region and blank region (the red boxes in (a)) as a function of potential at 40ºC and 50ºC *Te*. (c) ECL shadow image of cells with catalytic route in 1 mM $Ru(bpy)_3^{2+}$ and 10 mM TPrA. (d) The profiles of ECL contrast between cell region and blank region (the red boxes in (c)) as a function of potential at 40ºC and 50ºC *Te*. Scale bar (white) is 20 µm.

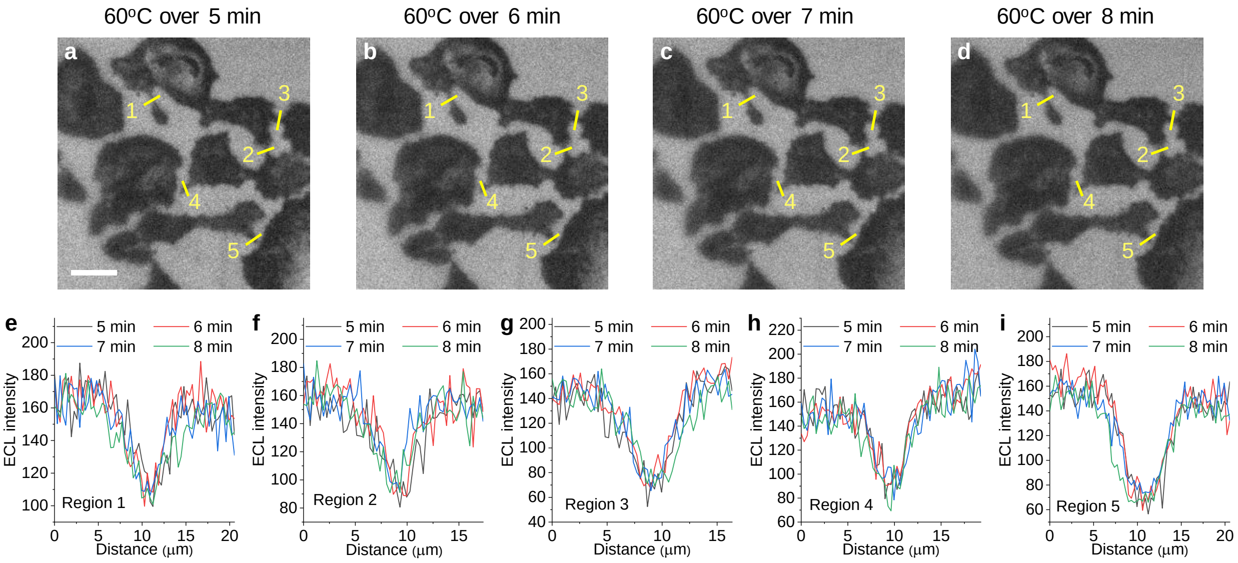

Figure S21. (a-d) ECL shadow images of cells under 60°C *Te* over 5 min, 6 min, 7 min and 8 min. The yellow lines indicate five subtle adherent cellular structures. (e-i) ECL profiles across five yellow lines, namely, subtle cellular structure regions in (a-d) at different time. The electrolyte contains 1 mM $Ru(bpy)_3^{2+}$ and 100 mM TPrA. Scale bar (white) is 30 μm.

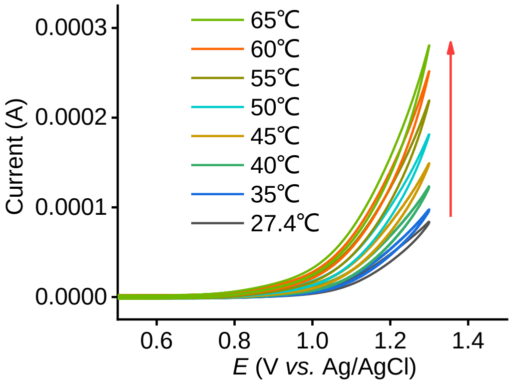

Figure S22. Anodic oxidation current of TPrA in CV scans at different *Te* from 27.4°C to 65°C. The electrolyte is 1 mM TPrA in 200 mM PBS (pH = 7.0). Work electrode is ITO.

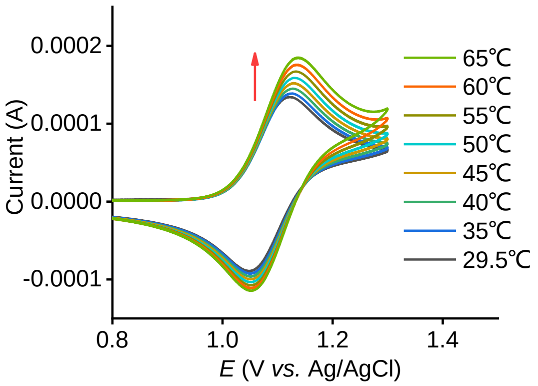

Figure S23. CV curves of $Ru(bpy)_3^{2+}$ at different *Te* from 29.5°C to 65°C. The electrolyte is 400 μM $Ru(bpy)_3^{2+}$ in 200 mM PBS (pH = 7.0). Work electrode is ITO.

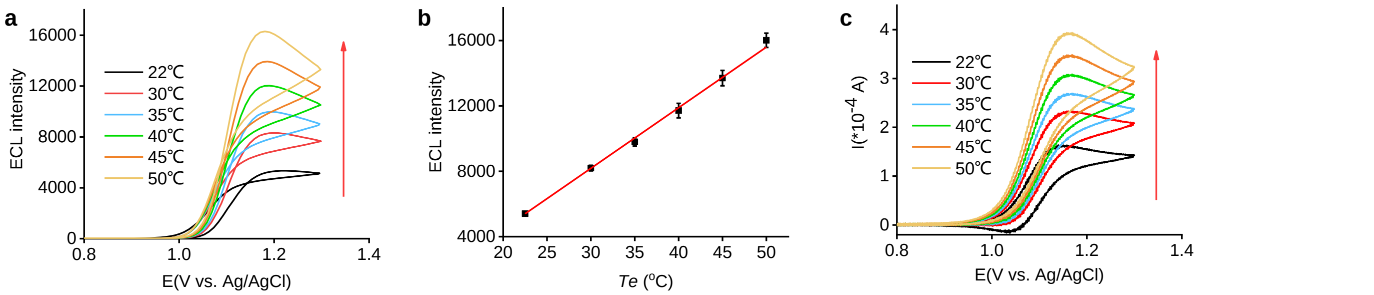

Figure S24. (a) ECL-potential curves with elevating *Te* from 22°C to 50°C. (b) Positively linear correlation of ECL peak intensity and *Te*. (c) The corresponding cyclic voltammetry curve with elevating *Te* from 22°C to 50°C. The electrolyte is 400 μM $Ru(bpy)_3^{2+}$ and 1 mM TPrA in 200 mM PBS (pH = 7.0). Work electrode is ITO.

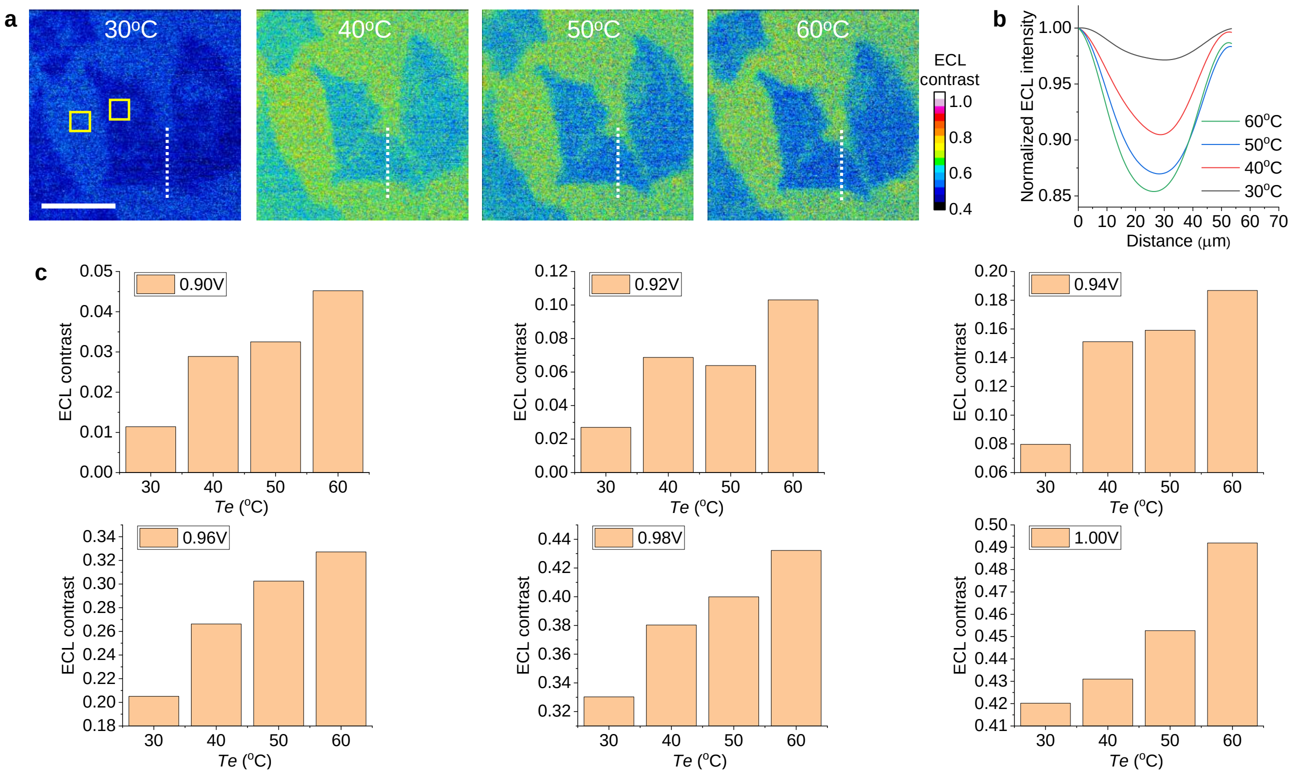

Figure S25. (a) ECL shadow images of adherent cells with elevating *Te* from 30°C to 60°C at 0.96 V potential. Scale bar (white) is 50 μm. (b) Normalized ECL profiles along the white dash line in (a), showing the increased ECL contrast with elevating *Te*. (c) ECL contrast of cell region versus blank region (two yellow boxes in (a)) with elevating *Te* from 30°C to 60°C. The setting potentials are 0.90 V, 0.92 V, 0.94 V, 0.96 V, 0.98 V and 1.00 V, respectively. The electrolyte is 1 mM $Ru(bpy)_3^{2+}$ and 10 mM TPrA in 200 mM PBS (pH = 7.0). Work electrode is ITO.

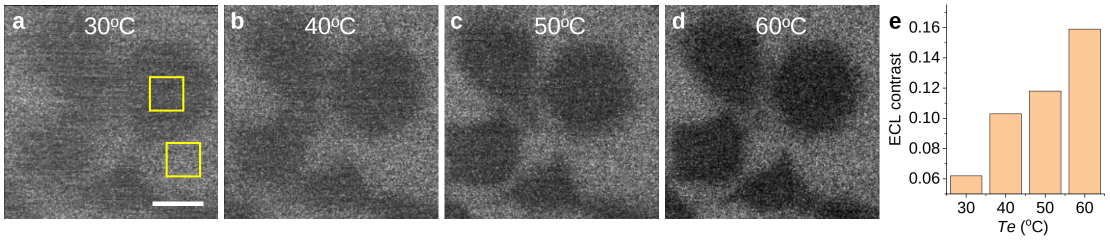

Figure S26. (a-d) ECL shadow images of adherent cells with elevating *Te* from 30°C to 60°C in low concentrations of TPrA (1 mM) and $Ru(bpy)_3^{2+}$ (50 μM). The applied voltage is 1.3 V. Scale bar (white) is 20 μm. (e) ECL contrast between cell region and blank region (two yellow boxes in (a)) with elevating *Te* from 30°C to 60°C.

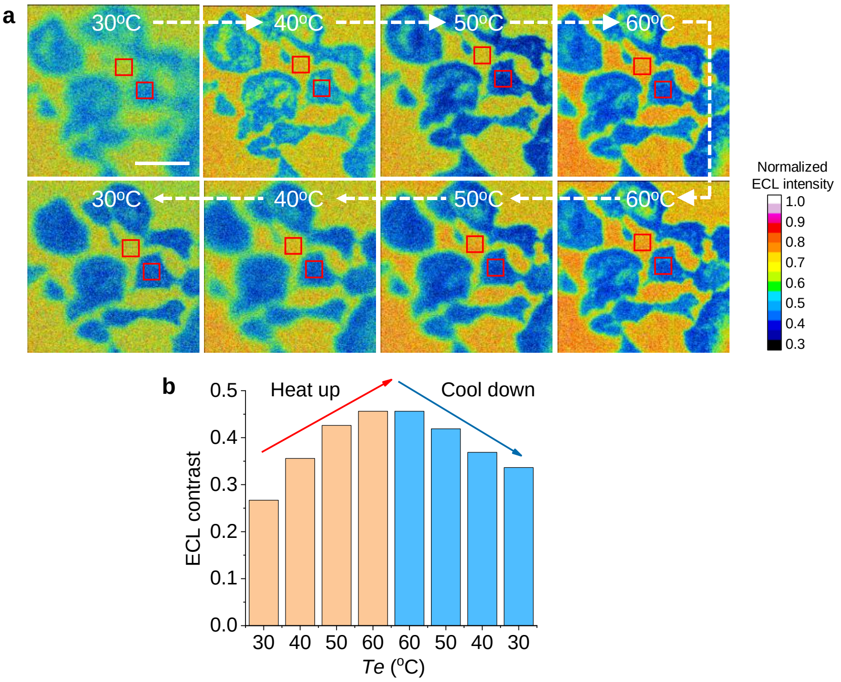

Figure S27. (a) ECL contrast of cell adherent regions showed a good reversibility with changing *Te* between 30°C and 60°C. The electrolyte contains 100 µM $Ru(bpy)_3^{2+}$ and 10 mM TPrA in 200 mM PBS (pH = 7.0). Scale bar (white) is 50 µm. (b) ECL contrast between cell region and blank region (two red boxed in (a)) with *Te* going up and down.

Experimental section

Chemicals and reagents

Unless specially illustrated, all the other chemicals and reagents used in this study were of analytical quality and were used as received without further purification. Tris-(2,2'-bipyridyl)dichlororuthenium(II) hexahydrate ($Ru(bpy)_3^{2+}$), tripropylamine (TPrA) were purchased from Sigma-Aldrich. Phosphate buffer solution (PBS, pH 7.4), Dulbecco's modified Eagle's medium (DMEM) with 10% fetal calf serum, trypsin and 4% paraformaldehyde solution were purchased from KeyGen BioTECH. A Hela cell line were purchased from the Institute of Cell Biology at the Chinese Academy of Sciences (Shanghai, P.R., China). Ultrapure water with a resistivity of 18.2 MΩ cm was produced by using a Milli-Q apparatus (Millipore) and used in the preparation of all solutions. PDMS was prepared by using Sylgard 184, Dow Corning (Midland, MI, USA).

Software

The image analysis and processing were performed by ImageJ and Origin 2018 software. The simulation was performed by COMSOL Multiphysics 5.6 software.

References

1 Miao, W., Choi, J. P. & Bard, A. J. Electrogenerated chemiluminescence 69: the tris(2,2'-bipyridine)ruthenium(II), ($Ru(bpy)_3^{(2+)}$)/tri-n-propylamine (TPrA) system revisited-a new route involving TPrA*⁺ cation radicals. *J. Am. Chem. Soc.* **124**, 14478-14485 (2002).

2 Ding, H. *et al.* Spatially Selective Imaging of Cell-Matrix and Cell-Cell Junctions by Electrochemiluminescence. *Angew. Chem. Int. Ed.* **60**, 11769-11773 (2021).

3 Guo, W., Zhou, P., Sun, L., Ding, H. & Su, B. Microtube Electrodes for Imaging the Electrochemiluminescence Layer and Deciphering the Reaction Mechanism. *Angew. Chem. Int. Ed.* **60**, 2089-2093 (2021).

4 Ma, C. *et al.* Dynamically imaging collision electrochemistry of single electrochemiluminescence nano-emitters. *Chem. Sci.* **9**, 6167-6175 (2018).

Author Contributions

C.M., Z.X., L.P.J. and J.J.Z. conceived the study. Z.X. and C.M. performed the experiments. Z.X., C.M. and X.G. built the microscope and analyzed the data. C.M., L.P.J. and J.J.Z. advised the manuscript. C.M., Z.X. and J.J.Z. wrote the paper.
